# Economic and biophysical limits to seaweed farming for climate change mitigation

**DOI:** 10.1038/s41477-022-01305-9

**Published:** 2022-12-23

**Authors:** Julianne DeAngelo, Benjamin T. Saenz, Isabella B. Arzeno-Soltero, Christina A. Frieder, Matthew C. Long, Joseph Hamman, Kristen A. Davis, Steven J. Davis

**Affiliations:** 1 Department of Earth System Science, University of California, Irvine, Irvine, CA USA; 2 Biota.earth, Berkeley, CA USA; 3 Department of Civil and Environmental Engineering, Stanford University, Stanford, CA USA; 4 Southern California Coastal Water Research Project, Costa Mesa, CA USA; 5 National Center for Atmospheric Research, Boulder, CO USA; 6 Earthmover, New York, NY USA; 7 Department of Civil and Environmental Engineering, University of California, Irvine, Irvine, CA USA

**Keywords:** Climate-change mitigation, Environmental economics, Climate-change mitigation

## Abstract

Net-zero greenhouse gas (GHG) emissions targets are driving interest in opportunities for biomass-based negative emissions and bioenergy, including from marine sources such as seaweed. Yet the biophysical and economic limits to farming seaweed at scales relevant to the global carbon budget have not been assessed in detail. We use coupled seaweed growth and technoeconomic models to estimate the costs of global seaweed production and related climate benefits, systematically testing the relative importance of model parameters. Under our most optimistic assumptions, sinking farmed seaweed to the deep sea to sequester a gigaton of CO_2_ per year costs as little as US$480 per tCO_2_ on average, while using farmed seaweed for products that avoid a gigaton of CO_2_-equivalent GHG emissions annually could return a profit of $50 per tCO_2_-eq. However, these costs depend on low farming costs, high seaweed yields, and assumptions that almost all carbon in seaweed is removed from the atmosphere (that is, competition between phytoplankton and seaweed is negligible) and that seaweed products can displace products with substantial embodied non-CO_2_ GHG emissions. Moreover, the gigaton-scale climate benefits we model would require farming very large areas (>90,000 km^2^)—a >30-fold increase in the area currently farmed. Our results therefore suggest that seaweed-based climate benefits may be feasible, but targeted research and demonstrations are needed to further reduce economic and biophysical uncertainties.

## Abstract (web summary)

Potential climate benefits of farming seaweed are large but sensitive to uncertain yields and competition with phytoplankton. Carbon removal by sinking seaweed is much costlier than avoiding emissions by substituting seaweed for land-based crops.

## Main

Reaching net-zero CO_2_ emissions will entail drastically reducing fossil fuel emissions and offsetting any residual emissions by removing carbon from the atmosphere (that is, negative emissions)^1–5^. Biomass-based technologies may help in both fronts by supplying carbon-neutral alternatives to fossil fuels^6,7^ and providing negative emissions via enhancement of natural sinks^8^ and/or bioenergy with carbon capture and storage^9^. However, numerous studies have questioned whether terrestrial biomass can provide either energy or negative emissions at the scales required in many climate mitigation scenarios, often owing to limited land and water resources^10–12^. This has driven surging interest in ocean-based carbon dioxide removal, including via cultivated macroalgae (seaweed), which would not require inputs of land or freshwater and might have environmental co-benefits (for example, see refs. 13–21). Seaweed products might also help to lower greenhouse gas emissions, for example by reducing methane emissions from ruminants^22^, and replacing fossil fuels^23^ and emissions-intensive agricultural products^24^.

Seaweed has been successfully farmed in some places for centuries, and used for food, animal feed, and in more modern times, cosmetics, medicine, fertilizer and biofuels^25–28^. Production of seaweed for food increased 6% per year in 2000–2018^29^ and harvest totalled ~1 million tons of carbon worldwide in 2018^29^. In comparison, climate scenarios that limit warming to 1.5 ° or 2 °C generally require more than 1 gigaton of carbon (that is >3.67 GtCO_2_) to be removed annually from the atmosphere in the year CO_2_ emissions reach net-zero^3^. To contribute to such climate goals, seaweed farming must therefore expand tremendously, and in turn contend with large uncertainties in the productivity of different types of seaweed in different places, the net costs of farming, the magnitude of emissions avoided or carbon sequestered, and the potential for undesirable ecological impacts. Recent studies of seaweed farming have examined localized opportunities and dynamics in particular regions^15,16,30^, made rough estimates of the global potential^13,14,31,32^ and modelled the Earth system response to gigaton-scale production^19^. Yet the productivity, costs and potential climate benefits of such farming are spatially heterogeneous and scale-dependent, and the key sensitivities and trade-offs important to investors and decision-makers have not been comprehensively evaluated. Here we use coupled biophysical and technoeconomic models to systematically assess the economic costs and potential climate benefits of seaweed farming, testing their sensitivity across large ranges in individual variables and comparing different product pathways.

Details of our analytic approach are described in Methods. In summary, we use outputs from a newly developed biophysical model (G-MACMODS)^33,34^ to estimate potential harvest of four different seaweed types (tropical red, tropical brown, temperate red and temperate brown; Supplementary Fig. 1) at a resolution of 1/12° (~9 km at the equator) globally. Nutrients are a key constraint on seaweed growth. G-MACMODS assumes that nitrogen is the limiting nutrient and we model two idealized scenarios: an ‘ambient’ nutrient scenario that computes growth on the basis of observed climatological surface nitrate concentrations, and a ‘limited nutrient’ scenario that computes growth rate on the basis of ambient nitrate concentrations but limits algal biomass increases so as not to exceed the magnitude of local natural (upward) nitrate supply as estimated by a high-resolution simulation of the Community Earth System Model^35^. On the basis of the simulated yields, we then calculate spatially explicit costs per ton of seaweed harvested and either costs per ton of greenhouse gas (GHG) emissions avoided (when used as food, feed or for biofuels) or costs per ton of carbon removed from the atmosphere as a carbon dioxide removal (CDR) strategy. Given the large uncertainty in technoeconomic parameters, we perform a Monte Carlo simulation with *n* = 5,000 for each nutrient scenario, assuming uniform distributions of each variable. Technoeconomic variables include (1) farming costs (for example, capital cost, harvest costs), (2) for carbon sequestration, the fraction of sunk seaweed carbon sequestered for >100 yr in the deep sea and (3) for GHG emissions mitigation, the net cost and net emissions of seaweed transported and converted into a product (Table 1; see Supplementary Tables 1 and 2 for listings of all variables and relevant sources). We test model sensitivity to seaweed yield by sampling from a normal distribution of seaweed yield uncertainty for each Monte Carlo simulation. Additionally, because seaweed draws carbon from the surface ocean dissolved inorganic carbon pool (which does not maintain instantaneous equilibrium with the atmosphere) and because large-scale seaweed farming can reduce natural carbon uptake by phytoplankton via nutrient competition, we include a variable representing the net efficiency of seaweed growth in reducing atmospheric CO_2_ (‘atmospheric removal fraction’; Supplementary Table 1). Our approach is predicated on large uncertainties associated with most of the variables we analyse, not only in the future but also the present (the relatively few costs reported in the literature are location- and/or species-specific), as well as our primary goal of informing future research by identifying relative differences, sensitivities and trade-offs that are robust across our simulations.

**Table 1:** Ranges of selected variables used in our technoeconomic analysis

Variable	Unit	Model range	Values reported in literature
Capital costs	US$ km^−2^ yr^−1^	10,000–1,000,000	929,676 (ref. 40) 550,000–950,000 (ref. 62) 375,910 (ref. 61) 210,580 (ref. 41)
Seeded line cost (includes hatchery costs)	$ m^−1^	0.05–1.45	1.38 (ref. 40) 0.13 (ref. 41)
Harvest costs (includes harvest labour, excludes harvest transport)	$ km^−2^ per harvest	120,000–400,000	381,265 (ref. 41) 138,000 (ref. 40)
Transport cost per ton of material (includes loading/unloading costs)	$ t^−1^ km^−1^	0.1–0.35	0.225 (ref. 40)
Transport emissions per ton of material	tCO_2_ t^−1^ km^−1^	0–0.000045	0.00003 (ref. 28)
Maintenance boat emissions	tCO_2_ km^−1^	0–0.0035	0.0023653 (calculated using methods from refs. 28, 56)
Atmospheric removal fraction	fraction	0.4–1	0.4–0.75 (ref. 46) 0.5 (global average, from preliminary experiment by authors using ref. 35 informed by ref. 15)
Seaweed market value for product end-use	$ tDW^−1^	400–800	Food: 500–800 (dried seaweed wholesale price from ref. 63) Feed: 400–500 (values per ton dry animal feed and soybean meal from refs. 40, 64, assuming direct replacement with dry seaweed) Fuel: 430 (dried seaweed price for bioethanol production, calculated on the basis of bioethanol yield per ton seaweed (0.25) and average of 2021–2022 historical E85 fuel prices ($3.76 per gasoline gallon equivalent, GGE) from ref. 65, modelled range 400–500) Not product-specific: 400 (dried seaweed market price of $400 tDW^−1^ from ref. 25)
GHG emissions avoided by replacement with seaweed product	tCO_2_-eq tDW^−1^	0.7–6.0	Food: 1–6 (considering global average emissions from GHGs per kcal for pulses, vegetables, fruits, oil crops and cereals, from ref. 24) Feed: 1–3.1 (considering global average emissions from GHGs per kcal for oil crops and cereals, ±50% uncertainty, from ref. 24) Fuel: 0.7–1 (assuming 3.2–3.5 tCO_2_ t^−1^ fossil fuel by fuel type from ref. 66 and 0.25 t bioethanol per tDW yield from ref. 59, and energy density equivalence conversions by fuel type)

## Results

### Seaweed production cost

**Fig. 1:**
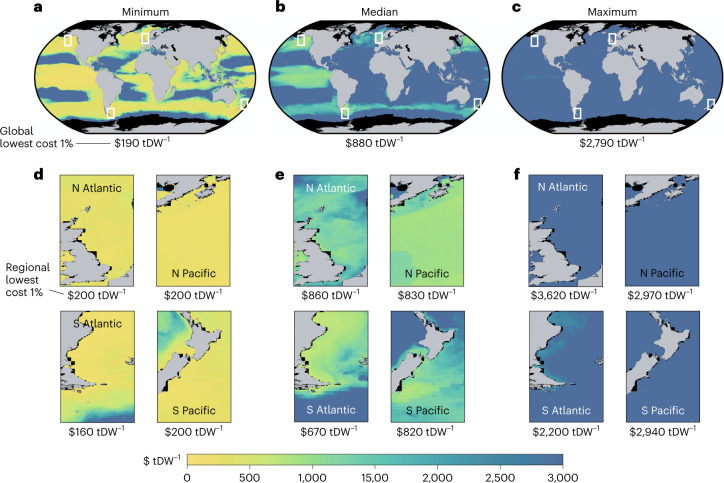
Seaweed production costs. **a**–**f**, Estimated seaweed production costs vary considerably depending on assumed costs of farming capital, seeded lines, labour and harvest (excluding transport of harvested seaweed). Across ambient-nutrient simulations, average farming cost in the 1% of global ocean areas with lowest cost ranges from $190 tDW^−1^ (**a**) to $2,790 tDW^−1^ (**c**), with a median of $880 tDW^−1^ (**b**). Regional insets (**d**–**f**) reveal small-scale features in particularly low-cost areas. Supplementary Fig. 2 shows maps for limited-nutrient simulations.

The maps in Fig. 1 show the range of modelled seaweed production costs (that is, US$ per ton of harvested dry weight (DW) before transport) in different regions under the ambient-nutrient scenario and assuming the most productive type of seaweed is grown in each grid cell (Supplementary Fig. 2 shows analogous costs for a limited-nutrient scenario). Minimum modelled costs (Fig. 1a,d) thus reflect high levels of seaweed growth (ambient nutrients) and very low assumed costs of farming, whereas the maximum costs in Supplementary Fig. 2c,f reflect lower levels of seaweed growth in most areas (limited nutrients) and high-end cost assumptions. Since our ability to accurately assess the role of nutrient constraints as a determinant of yield is a major driver of total uncertainty in cost, our results are thereby likely to encompass a wide range of outlooks, including substantial future reductions in farming costs related to technological breakthroughs, returns to scale and boosted productivity (for example, autonomous farms, depth cycling, artificial upwelling and offshore integrated multitrophic aquaculture^36,37^).

Although the spread in average cost in the 1% of ocean area where costs are lowest (labels beneath each panel) ranges from $190 to $2,790 per ton of dry weight (tDW) seaweed yield, regional patterns of production costs are relatively consistent across cost simulations (Fig. 1). For example, the equatorial Pacific, Gulf of Alaska and southeastern edge of South America are consistently among the lowest cost areas to produce seaweed (yellow and green shading in Fig. 1), and there are large swaths of ocean that cannot produce seaweed for <$2,000 tDW^−1^ in any case (areas shaded blue in Fig. 1). These patterns reflect the combination of seaweed productivity and the associated number of harvests (Supplementary Figs. 3 and 4, respectively). Higher harvest costs can erode the cost advantage of highly productive areas: for example, despite having much lower seaweed yields per unit area, the North Pacific’s lower harvest costs lead to production costs that are often similar to those in the Equatorial Pacific (Fig. 1 and Supplementary Fig. 3). Moreover, because transportation of harvested seaweed is not included in the at-farm production costs but rather in the post-cultivation costs (Methods), some areas of open ocean far from ports have low at-farm production costs. On average, the costs of seeded line, total harvest costs and capital costs (including mooring costs) dominate total production costs, representing 56 (32–92)%, 19 (4–38)% and 17(3–33)% across seaweed types, respectively (Supplementary Fig. 5).

Finally, since global seaweed yield is reduced in simulations that limit nutrient availability to natural vertical nutrient fluxes, the production costs in the 1% of ocean area with the lowest cost are much higher ($350–$7,150 tDW^−1^; Supplementary Fig. 2) than in simulations in which seaweed is allowed to use all ambient nutrients. This suggests that without methods to enhance nutrient availability (for example, depth cycling, artificial upwelling, or offshore integrated multitrophic aquaculture^36,37^), limiting seaweed yields to maintain surface ocean nutrient levels might be cost-prohibitive except in the most optimistic technoeconomic scenarios.

### Net cost of climate benefits

**Fig. 2:**
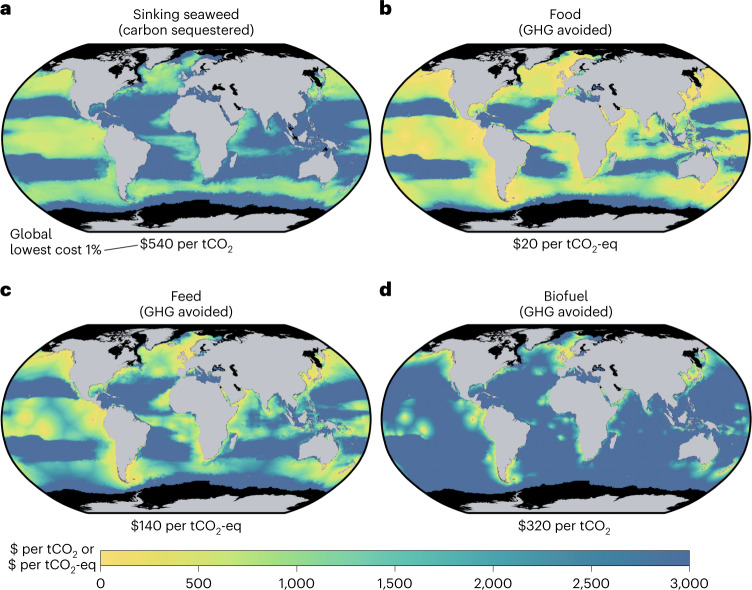
Net cost of potential seaweed climate benefits. **a**–**d**, Costs of using farmed seaweed to sequester carbon or avoid GHG emissions vary in space according to estimated production costs as well as spatially explicit differences in the costs and net emissions of transportation, sinking or conversion, and replacement of conventional market alternatives with seaweed products. Differentiation between seaweed product groups (**b**–**d**) is based on emissions avoided by seaweed products and market value for each product type. Maps show costs when propagating the most optimistic assumptions (5th percentile costs) from ambient-nutrient simulations. Average cost in the 1% of global ocean areas with lowest cost ranges from $20 per tCO_2_-eq avoided when seaweed is used for food (**b**) to $540 per tCO_2_ sequestered by sinking seaweed (**a**). Supplementary Figs. 6 and 7 show maps for limited-nutrient simulations and median costs, respectively.

The maps in Fig. 2 show net costs of different climate benefits from farmed seaweed. We choose to show costs when propagating the most optimistic assumptions (5th percentile costs) from ambient-nutrient simulations to reflect low-cost results that might be achieved with economies of scale (Supplementary Figs. 6 and 7 show results under limited nutrients and for median net costs, respectively). We define the cost to sequester carbon via sinking seaweed as the $ per tCO_2_ removed from the atmosphere for at least 100 yr, assuming no other economic value. In contrast, costs of emissions avoided by using produced seaweed for food, feed or biofuel are given in units of $ per tCO_2_-eq and in each case reflect seaweed production, transportation and conversion costs, and the product’s market value as well as the CO_2_-equivalent GHG emissions (CH_4_ and N_2_O assuming Global Warming Potential (GWP)_100_) displaced by the product net of any emissions related to transportation and processing (Methods). When calculating GHG emissions avoided, we assume that products made from seaweed can directly replace conventional food (pulses, vegetables, fruits, oil crops and cereals), feed (oil crops and cereals) and fuels, thereby avoiding GHG emissions from industrial agriculture practices or CO_2_ emissions from fossil fuel combustion^24^. For example, if seaweed is used for food and replaces some amount of vegetables in a person’s diet, then the GHG emissions associated with the production of those vegetables that the seaweed replaces are counted as avoided emissions.

In the lowest-cost 1% ocean areas, the average cost is much higher per ton of carbon sequestered by sinking seaweed ($540 per tCO_2_) than per ton of CO_2_-eq emissions avoided, regardless of whether the seaweed is used for food ($20 per tCO_2_-eq), animal feed ($140 per tCO_2_-eq) or biofuel ($320 per tCO_2_-eq). The substantial cost difference between sequestration by sinking and emissions avoided by products is most influenced by the products’ market value and the potential to avoid non-CO_2_ GHGs, despite the higher cost and emissions required to transport harvested seaweed to port.

In particular, the non-CO_2_ GHG emissions that could be avoided by using seaweed for either food consumed by humans or feed consumed by animals effectively multiply the potential climate benefits of a ton of seaweed carbon, whereas the climate benefits of either sinking or converting seaweed to biofuels are constrained by the carbon present in the seaweed itself. Yet carbon sequestration is nonetheless favoured in some locations given the high costs of transporting seaweed back to the nearest port (for example, areas of the equatorial Pacific that are shaded yellow and green in Fig. 2a and blue in Fig. 2c; see also Supplementary Fig. 8).

### Key sensitivities

**Fig. 3:**
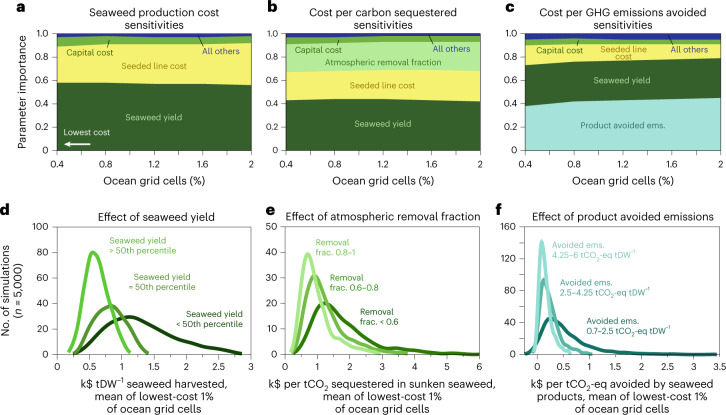
Key cost sensitivities of seaweed production and climate benefits. **a**–**c**, Across Monte Carlo simulations in the 2% of ocean grid cells where costs are lowest, estimated seaweed production cost is especially sensitive to the seaweed yield amount and seeded line cost (**a**), whereas costs of carbon sequestration (**b**) and GHG emissions avoided (**c**) are strongly influenced by the fraction of seaweed carbon that corresponds to an equivalent amount removed from the atmosphere and the assumed emissions avoided by seaweed products, respectively, in addition to seaweed yield and seeded line cost. **d**–**f**, Kernel density plots for the most important parameters in the cheapest 1% ocean areas, showing that the lowest production and climate benefit costs depend upon seaweed yield being at or above the median of potential seaweed yields (**d**), an assumed atmospheric removal fraction of >0.6–0.8 (**e**) and avoided emissions >2.5 tCO_2_-eq tDW^−1^ (**f**). Supplementary Fig. 9 shows cost sensitivities in limited-nutrient simulations.

Figure 3 shows the relative importance of all variables in generating spread in our Monte Carlo estimates of production costs and net costs of climate benefits, focusing on the lowest-cost areas (Supplementary Fig. 9 shows the same results for limited-nutrient simulations). These results emphasize which variables are most important to achieving very low costs. Low production costs are most sensitive to seaweed yields, followed by the cost of seeded line (secondary line with seaweed seedlings that is wrapped around a structural rope, or nets for some temperate red seaweeds; yellow in Fig. 3a) and capital costs (for example, boats, harvest machines, buoys, anchors and other lines; green in Fig. 3a). Together, seaweed yield and seeded line cost account for >89% of the uncertainty in production costs in the places where costs are lowest, and costs are never below $400 tDW^−1^ in simulations where seeded line is assumed to cost >$1 m^−1^.

Costs of carbon sequestered are quite sensitive to production costs (including all parameters shown in Fig. 3a), but the most important parameter aside from production costs and yield is the fraction of the seaweed carbon that corresponds to equivalent carbon removal from the atmosphere (light green in Fig. 3b). Although this fraction has generally been assumed to be 1, recent studies have shown that air–sea fluxes of CO_2_ may not keep pace with carbon uptake by growing seaweed and, among other mechanisms that reduce efficiency, nutrient competition from farmed seaweed may diminish natural carbon uptake and export accomplished by phytoplankton^15,19^. The atmospheric removal fraction accounts for >24% of the variation in sequestration costs in the places where costs are lowest, and costs are never below $400 per tCO_2_ sequestered unless the removal fraction is assumed to be >0.6 (Fig. 3b,e).

Our estimates of cost per GHG emissions avoided are most sensitive to the assumed magnitude of CO_2_-equivalent emissions avoided by a seaweed product (light blue in Fig. 3c). The product-avoided emissions account for >38% of the variation in costs per emissions avoided in the places where costs are lowest, and costs are never more than $700 per tCO_2_-eq avoided in simulations where the product-avoided emissions are assumed to be >4.25 tCO_2_-eq tDW^−1^ seaweed (Fig. 3c,f). Yet production costs remain important, and low costs of emissions avoided (<$200 per tCO_2_-eq) can be achieved even when the avoided emissions are <1 tCO_2_-eq tDW^−1^ if seaweed production costs are very low (Fig. 3c,f).

### Costs and benefits of large-scale seaweed farming

**Fig. 4:**
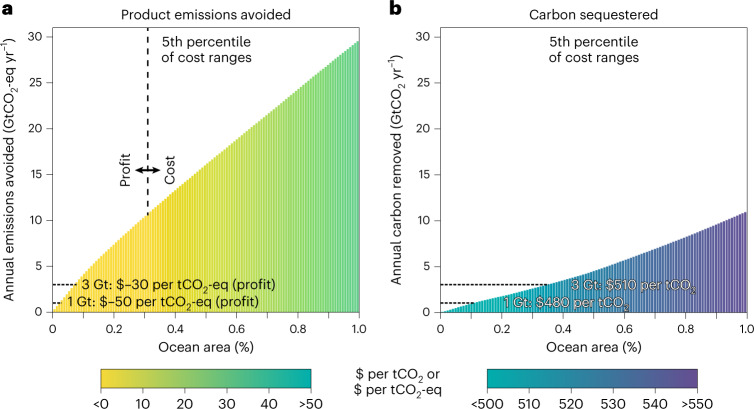
Cumulative potential climate benefits of large-scale seaweed farming. **a**,**b**, Total GHG emissions avoided (**a**) or carbon sequestered (**b**) each year could reach gigaton scales if seaweed were farmed over large areas of the ocean. Bars show the potential climate benefits as a function of the lowest-cost ocean area (0.1% of ocean area is roughly 360,000 km^2^, nearly the area of Germany and 130 times the total area of current seaweed farms), and colours indicate the average cost (or profit) per tCO_2_-eq emissions avoided or tCO_2_ sequestered using optimistically low net costs (5th percentile) from ambient-nutrient simulations. Supplementary Figs. 10 and 11 show cumulative potential and costs at the median and in limited-nutrient simulations.

Figure 4 shows the cumulative potential of GHG emissions avoided or carbon sequestered in the 1% of ocean areas with the lowest costs, shaded with costs per ton on the basis of the 5th percentile of 5,000 ambient nutrient–cost simulations (that is, reflecting optimistically high seaweed yield, low farming costs and large climate benefits from replacement of agricultural products; Supplementary Figs. 10 and 11 show results for median costs and limited-nutrient scenario). No matter the scenario or percentile, in the 1% of areas with the lowest costs, the costs per ton of CO_2_ sequestered are always higher than the costs per ton of CO_2_-eq emissions avoided. In the optimistic case depicted in Fig. 4, 1 Gt of CO_2_-eq emissions might be avoided or 1 Gt of CO_2_ sequestered by farming 0.025% and 0.110% of lowest-cost ocean areas, respectively (roughly 90,000 km^2^ and 400,000 km^2^ or close to the areas of Portugal and Zimbabwe, respectively), at an average profit of $50 per tCO_2_-eq emissions avoided or at an average cost of $480 per tCO_2_ sequestered. In limited-nutrient simulations with optimistic cost assumptions (Supplementary Fig. 11a,b), the lowest-cost ocean area that might be required to reach 1 GtCO_2_-eq avoided emissions or 1 GtCO_2_ sequestered annually is 0.035% and 0.100% for avoided emissions and sequestration, respectively, or roughly 130,000 km^2^ and 360,000 km^2^, with associated costs of $30 per tCO_2_-eq avoided and $830 per tCO_2_ sequestered. Average costs at the median of Monte Carlo simulations for both nutrient scenarios rise substantially to $110–310 per tCO_2_-eq emissions avoided or $1,120–$2,090 per tCO_2_ sequestered, respectively (Supplementary Figs. 10 and 11a,b). These costs increase to $140–420 per tCO_2_-eq at 3 GtCO_2_-eq avoided and to $1,190–2,280 per tCO_2_ at 3 GtCO_2_ sequestered annually, requiring ocean areas of 0.085–0.100% and 0.285–0.410% for avoided emissions and sequestration, respectively (roughly 310,000–360,000 km^2^ and 1,030,000–1,480,000 km^2^). Moreover, climate benefits increase approximately linearly with area up to 1% of ocean area, reaching totals of >29 GtCO_2_-eq avoided or >9 GtCO_2_ sequestered annually in the ambient-nutrient simulations and >19 GtCO_2_-eq avoided or >8 GtCO_2_ sequestered annually in the limited-nutrient simulations.

Supplementary Fig. 12 shows the locations of the lowest cost areas in Fig. 4, which, for sequestration, are concentrated in the equatorial Pacific and Gulf of Alaska, and for avoided emissions products include additional areas offshore of Argentina, the Korean Peninsula and New Zealand as well as areas of the North and Norwegian Seas. Importantly, we estimate that perhaps 10–15% of lowest cost areas for sequestration and 40–45% of lowest cost areas for avoided emissions are either in highly trafficked shipping lanes or part of existing marine protected areas (Methods), which could present challenges for seaweed farming in these areas.

Despite being a small percentage of global ocean area, farming 0.025% of the global ocean area (~90,000 km^2^) would represent over a 30-fold increase in the area of current seaweed farming (~2,700 km^2^; refs. 29, 38, 39). Thus, producing seaweed in the lowest cost areas to reach 1 GtCO_2_-eq of emissions avoided or 1 GtCO_2_ sequestered by 2050 would entail increasing the area farmed by roughly 12% or 18% per year, respectively, compared with the 2000–2018 seaweed farming industrial growth rate of 6%^29^. Achieving the same level of climate benefits from seaweed by 2030 increases the implied expansion rate of farms to roughly 42% or 64% per year for emissions avoided or carbon sequestered, respectively. Note that these areas and industry growth rates reflect the minimum that might be required for gigaton-scale climate impact, since the ambient-nutrient scenario assumes that all surface ocean nutrients are available for seaweed growth. In the limited-nutrient scenario, reaching 1 GtCO_2_-eq of emissions avoided in the lowest-cost areas by 2050 might require ~130,000 km^2^, which would represent a nearly 50-fold increase in the area currently farmed and would entail a 14% annual growth rate.

## Discussion

Our results suggest that it might be possible to sequester >1 GtCO_2_ at costs as low as $480 per tCO_2_ if nearly all seaweed carbon corresponds directly to an amount of CO_2_ removed from the atmosphere, production costs are reduced to near the lowest published costs^40,41^ (for example, seeded line and capital costs of < $0.40 m^−1^ and $3,300 ha^−1^, respectively), and/or seaweed yields are high (for example, >6,000 tDW km^−2^ for tropical reds and >2,000 tDW km^−2^ for temperate browns). Nonetheless, $480 per tCO_2_ is comparable to the $500–600 t per CO_2_ costs of direct air capture (DAC) reported by the company Climeworks^42^ (but much more than the $94–$232 DAC costs estimated in ref. 43). Sequestration costs also rise sharply if the assumed atmospheric removal fraction or seaweed yield decreases or if production costs increase (Supplementary Figs. 7 and 10, and Fig. 3e). In comparison, >1 GtCO_2_-eq emissions might be avoided at a profit of $50 per tCO_2_-eq if similarly low production costs are achieved and seaweed products avoid emissions of >3 tCO_2_-eq tDW^−1^ (for example, by displacing vegetables, legumes, or soy from some regions). Although the cost per emission avoided is typically higher if seaweed is instead used for biofuels (Fig. 2, and Supplementary Figs. 6 and 7), such fuels may command a substantial ‘green premium’ as countries seek to decarbonize aviation and long-distance transportation of freight^4,7,44,45^.

Although it is thus conceivable that farmed seaweed could feasibly deliver globally relevant climate benefits, our modelling and cost estimates are subject to important caveats and limitations. First, modelled economic parameter ranges are broad, spanning a relatively small number of divergent data points from publicly available datasets and scientific literature. In some cases, these ranges were extended downward to reflect potential future cost reductions that were not represented by existing data. Better constraining these cost ranges for both current and future scenarios would improve the model and reduce uncertainty. Similarly, future work could analyse in greater detail the specific types and scale of agricultural or energy product that might be displaced by seaweed and their GHG emissions. Although the relative benefits of avoiding different GHG emissions versus sequestering carbon for different periods of time are beyond the scope of our analysis, they may be important to investors and decision-makers. For example, in many potentially low-cost seaweed production regions, the time scale of sunk carbon that remains ‘sequestered’ in the deep ocean is less than 100 yr^8^; if CDR accounting requires multi-century sequestration, the cost of seaweed-based CDR may become prohibitively high.

There are also large sources of uncertainty that deserve further exploration in the future. For example, we find that estimated costs per ton of CO_2_-eq emissions avoided or CO_2_ sequestered are highly sensitive to both the nutrient scenario (ambient vs. limited nutrients, Fig. 2 and Supplementary Fig. 6) and yield uncertainty within each nutrient scenario. Nutrient reallocation from competition between farmed seaweed and phytoplankton is also a critical dynamic that warrants analysis in the context of a fully coupled earth system model, since farming seaweed at gigaton scales would probably diminish natural carbon uptake by phytoplankton and therefore reduce the net drawdown of atmospheric CO_2_^46^. Moreover, our climate benefit calculations do not include particulate seaweed biomass that may be exported to the deep sea before harvest (analogous to sinking ~5% of the harvested biomass^21^), and we also do not consider any potential non-CO_2_ GHG emissions from the seaweed cultivation process. The G-MACMODS model also assumes that nitrogen is the limiting macronutrient for seaweed growth (and micronutrients are supplemented by farming techniques), and while nitrogen limits production in large parts of the ocean, phosphorus might be more limiting in some regions. Finally, we must continue to evaluate potential consequences to ocean ecosystems and biogeochemical cycles before seriously considering farming and/or sinking gigatons of seaweed^19^.

Despite these uncertainties and limitations, our analysis supports continued research, development and demonstration of the potential for seaweed farming to produce meaningful climate benefits. Specifically, our model highlights the most important targets for research and innovation. Biophysical factors such as death (including disease, pests, weather events) and exudation rates are not well-established and may substantially alter projected seaweed yields^33^; regional biogeochemical and Earth system feedbacks could similarly undermine the efficacy of sinking seaweed carbon; and low or narrow demand for seaweed products could limit the potential to offset land-use and fossil GHG emissions. Finally, although some seaweed innovators are focused on farm designs that reduce labour and transportation costs, our results suggest that the keys to maximizing yield with low production costs are seeded line and basic farm equipment such as boats, buoys and anchors. However, even if seed and capital costs are minimized, seaweed CDR seems likely to be more expensive than alternatives such as direct air capture, and it is not clear that there are viable and large markets for seaweed products. These factors, combined with the challenges inherent in verification and monitoring as well as the potential for ecosystem disruption, suggest that expansion of seaweed cultivation should be approached with caution. The outlook for a massive scale-up of seaweed climate benefits is thus decidedly murky, but our findings can help direct research, investments and decision making to clear the waters.

## Methods

### Monte Carlo analysis

Seaweed production costs and net costs of climate benefits were estimated on the basis of outputs of the biophysical and technoeconomic models described below. The associated uncertainties and sensitivities were quantified by repeatedly sampling from uniform distributions of plausible values for each cost and economic parameter (*n* = 5,000 for each nutrient scenario from the biophysical model, for a total of *n* = 10,000 simulations; see Supplementary Figs. 14 and 15)^47–52^. Parameter importance across Monte Carlo simulations (Fig. 3 and Supplementary Fig. 9) was determined using decision trees in LightGBM, a gradient-boosting machine learning framework.

### Biophysical model

G-MACMODS is a nutrient-constrained, biophysical macroalgal growth model with inputs of temperature, nitrogen, light, flow, wave conditions and amount of seeded biomass^30,53^, that we used to estimate annual seaweed yield per area (either in tons of carbon or tons of dry weight biomass per km^2^ per year)^33,34^. In the model, seaweed takes up nitrogen from seawater, and that nitrogen is held in a stored pool before being converted to structural biomass via growth^54^. Seaweed biomass is then lost via mortality, which includes breakage from variable ocean wave intensity. The conversion from stored nitrogen to biomass is based on the minimum internal nitrogen requirements of macroalgae, and the conversion from biomass to units of carbon is based on an average carbon content of macroalgal dry weight (~30%)^55^. The model accounts for farming intensity (sub-grid-scale crowding) and employs a conditional harvest scheme, where harvest is optimized on the basis of growth rate and standing biomass^33^.

The G-MACMODS model is parameterized for four types of macroalgae: temperate brown, temperate red, tropical brown and tropical red. These types employed biophysical parameters from genera that represent over 99.5% of present-day farmed macroalgae (*Eucheuma*, *Gracilaria*, *Kappahycus*, *Sargassum*, *Porphyra*, *Saccharina*, *Laminaria*, *Macrocystis*)^39^. Environmental inputs were derived from satellite-based and climatological model output mapped to 1/12-degree global resolution, which resolves continental shelf regions. Nutrient distributions were derived from a 1/10-degree resolution biogeochemical simulation led by the National Center for Atmospheric Research (NCAR) and run in the Community Earth System Model (CESM) framework^35^.

Two nutrient scenarios were simulated with G-MACMODS and evaluated using the technoeconomic model analyses described below: the ‘ambient nutrient’ scenario where seaweed growth was computed using surface nutrient concentrations without depletion or competition, and ‘limited nutrient’ simulations where seaweed growth was limited by an estimation of the nutrient supply to surface waters (computed as the flux of deep-water nitrate through a 100 m depth horizon). For each Monte Carlo simulation in the economic analysis, the technoeconomic model randomly selects either the 5th, 25th, 50th, 75th or 95th percentile G-MACMODS seaweed yield map from a normal distribution to use as the yield map for that simulation. Figures and numbers reported in the main text are based on the ambient-nutrient scenario; results based on the limited-nutrient scenario are shown in Supplementary Figures.

### Technoeconomic model

An interactive web tool of the technoeconomic model is available at https://carbonplan.org/research/seaweed-farming.

We estimated the net cost of seaweed-related climate benefits by first estimating all costs and emissions related to seaweed farming, up to and including the point of harvest at the farm location, then estimating costs and emissions related to the transportation and processing of harvested seaweed, and finally estimating the market value of seaweed products and either carbon sequestered or GHG emissions avoided.

#### Production costs and emissions

Spatially explicit costs of seaweed production ($ tDW^−1^) and production-related emissions (tCO_2_ tDW^−1^) were calculated on the basis of ranges of capital costs ($ km^−2^), operating costs (including labour, $ km^−2^), harvest costs ($ km^−2^) and transport emissions per distance travelled (tCO_2_ km^−1^) in the literature (Table 1, Supplementary Tables 1 and 2); annual seaweed biomass (tDW km^−2^, for the preferred seaweed type in each grid cell), line spacing and number of harvests (species-dependent) from the biophysical model; as well as datasets of distances to the nearest port (km), ocean depth (m) and significant wave height (m).

Capital costs were calculated as:

1 $$c_{cap} = c_{capbase} + \left( {c_{capbase} \times \left( {k_d + k_w} \right)} \right) + c_{sl}$$

where *c*_cap_ is the total annualized capital costs per km^2^, *c*_capbase_ is the annualized capital cost per km^2^ (for example, cost of buoys, anchors, boats, structural rope) before applying depth and wave impacts, *k*_d_ and *k*_w_ are the impacts of depth and waviness on capital cost, respectively, each expressed as a multiplier between 0 and 1 modelled using our Monte Carlo method and applied only to grid cells with depth >500 m and/or significant wave height >3 m, respectively, and *c*_sl_ is the total annual cost of seeded line calculated as:

2 $$c_{sl} = c_{slbase} \times p_{sline}$$

where *c*_slbase_ is the cost per metre of seeded line, and *p*_sline_ is the total length of line per km^2^, based on the optimal seaweed type grown in each grid cell.

Operating and maintenance costs were calculated as:

3 $$c_{op} = c_{ins} + c_{lic} + c_{lab} + c_{opbase}$$

where *c*_op_ is the total annualized operating and maintenance costs per km^2^, *c*_ins_ is the annual insurance cost per km^2^, *c*_lic_ is the annual cost of a seaweed aquaculture license per km^2^, *c*_lab_ is the annual cost of labour excluding harvest labour, and *c*_opbase_ is all other operating and maintenance costs.

Harvest costs were calculated as:

4 $$c_{harv} = c_{harvbase} \times n_{harv}$$

where *c*_harv_ is the total annual costs associated with harvesting seaweed per km^2^, *c*_harvbase_ is the cost per harvest per km^2^ (including harvest labour but excluding harvest transport), and *n*_harv_ is the total number of harvests per year.

Costs associated with transporting equipment to the farming location were calculated as:

5 $$c_{eqtrans} = c_{transbase} \times m_{eq} \times d_{port}$$

where *c*_eqtrans_ is total annualized cost of transporting equipment, *c*_transbase_ is the cost to transport 1 ton of material 1 km on a barge, *m*_eq_ is the annualized equipment mass in tons and *d*_port_ is the ocean distance to the nearest port in km.

The total production cost of growing and harvesting seaweed was therefore calculated as:

6 $$c_{prod} = \frac{{\left( {c_{cap}} \right) + \left( {c_{op}} \right) + \left( {c_{harv}} \right) + (c_{eqtrans})}}{{s_{dw}}}$$

where *c*_prod_ is total annual cost of seaweed production (growth + harvesting), *c*_cap_ is as calculated in equation (1), *c*_op_ is as calculated in equation (3), *c*_harv_ is as calculated in equation (4), *c*_eqtrans_ is as calculated in equation (5) and *s*_dw_ is the DW of seaweed harvested annually per km^2^.

Emissions associated with transporting equipment to the farming location were calculated as:

7 $$e_{eqtrans} = e_{transbase} \times m_{eq} \times d_{port}$$

where *e*_eqtrans_ is the total annualized CO_2_ emissions in tons from transporting equipment, *e*_transbase_ is the CO_2_ emissions from transporting 1 ton of material 1 km on a barge, *m*_eq_ is the annualized equipment mass in tons and *d*_port_ is the ocean distance to the nearest port in km.

Emissions from maintenance trips to/from the seaweed farm were calculated as:

8 $$e_{mnt} = \left( {\left( {2 \times d_{port}} \right) \times e_{mntbase} \times \left( {\frac{{n_{mnt}}}{{a_{mnt}}}} \right)} \right) + (e_{mntbase} \times d_{mnt})$$

where *e*_mnt_ is total annual CO_2_ emissions from farm maintenance, *d*_port_ is the ocean distance to the nearest port in km, *n*_mnt_ is the number of maintenance trips per km^2^ per year, *a*_mnt_ is the area tended to per trip, *d*_mnt_ is the distance travelled around each km^2^ for maintenance and *e*_mntbase_ is the CO_2_ emissions from travelling 1 km on a typical fishing maintenance vessel (for example, a 14 m Marinnor vessel with 2 × 310 hp engines) at an average speed of 9 knots (16.67 km h^−1^), resulting in maintenance vessel fuel consumption of 0.88 l km^−1^ (refs. 28, 56).

Total emissions from growing and harvesting seaweed were therefore calculated as:

9 $$e_{prod} = \frac{{(e_{eqtrans}) + (e_{mnt})}}{{s_{dw}}}$$

where *e*_prod_ is total annual emissions from seaweed production (growth + harvesting), *e*_eqtrans_ is as calculated in equation (7), *e*_mnt_ is as calculated in equation (8) and *s*_dw_ is the DW of seaweed harvested annually per km^2^.

#### Market value and climate benefits of seaweed

Further transportation and processing costs, economic value and net emissions of either sinking seaweed in the deep ocean for carbon sequestration or converting seaweed into usable products (biofuel, animal feed, pulses, vegetables, fruits, oil crops and cereals) were calculated on the basis of ranges of transport costs ($ tDW^−1^ km^−1^), transport emissions (tCO_2_-eq t^−1^ km^−1^), conversion cost ($ tDW^−1^), conversion emissions (tCO_2_-eq tDW^−1^), market value of product ($ tDW^−1^) and the emissions avoided by product (tCO_2_-eq tDW^−1^) in the literature (Table 1). Market value was treated as globally homogeneous and does not vary by region. Emissions avoided by products were determined by comparing estimated emissions related to seaweed production to emissions from non-seaweed products that could potentially be replaced by seaweed (including non-CO_2_ greenhouse gas emissions from land use)^24^. Other parameters used are distance to nearest port (km), water depth (m), spatially explicit sequestration fraction (%)^57^ and distance to optimal sinking location (km; cost-optimized for maximum emissions benefit considering transport emissions combined with spatially explicit sequestration fraction; see ‘Distance to sinking point calculation’ below). Each Monte Carlo simulation calculated the cost of both CDR via sinking seaweed and GHG emissions mitigation via seaweed products.

For seaweed CDR, after the seaweed is harvested, it can either be sunk in the same location that it was grown, or be transported to a more economically favourable sinking location where more of the seaweed carbon would remain sequestered for 100 yr (see ‘Distance to optimal sinking point’ below). Immediately post-harvest, the seaweed still contains a large amount of water, requiring a conversion from dry mass to wet mass for subsequent calculations^33^:

10 $$s_{ww} = \frac{{s_{dw}}}{{0.1}}$$

where *s*_ww_ is the annual wet weight of seaweed harvested per km^2^ and *s*_dw_ is the annual DW of seaweed harvested per km^2^.

The cost to transport harvested seaweed to the optimal sinking location was calculated as:

11 $$c_{swtsink} = c_{transbase} \times d_{sink} \times s_{ww}$$

where *c*_swtsink_ is the total annual cost to transport harvested seaweed to the optimal sinking location, *c*_transbase_ is the cost to transport 1 ton of material 1 km on a barge, *d*_sink_ is the distance in km to the economically optimized sinking location and *s*_ww_ is the annually harvested seaweed wet weight in t km^−2^ as in equation (10).

The costs associated with transporting replacement equipment (for example, lines, buoys,

anchors) to the farming location and hauling back used equipment at the end of its assumed lifetime (1 yr for seeded line, 5–20 yr for capital equipment by equipment type) in the sinking CDR pathway were calculated as:

12 $$c_{eqtsink} = \left( {c_{transbase} \times \left( {2 \times d_{sink}} \right) \times m_{eq}} \right) + (c_{transbase} \times d_{port} \times m_{eq})$$

where *c*_eqtsink_ is the total annualized cost to transport both used and replacement equipment, *c*_transbase_ is the cost to transport 1 ton of material 1 km on a barge, *m*_eq_ is the annualized equipment mass in tons, *d*_sink_ is the distance in km to the economically optimized sinking location and *d*_port_ is the ocean distance to the nearest port in km. We assumed that the harvesting barge travels from the farming location directly to the optimal sinking location with harvested seaweed and replaced (used) equipment in tow (including used seeded line and annualized mass of used capital equipment), sinks the harvested seaweed, returns to the farm location and then returns to the nearest port (see Supplementary Fig. 16). These calculations assumed the shortest sea-route distance (see Distance to optimal sinking point).

The total value of seaweed that is sunk for CDR was therefore calculated as:

13 $$v_{sink} = \frac{{\left( {v_{cprice} - \left( {c_{swtsink} + c_{eqtsink}} \right)} \right)}}{{s_{dw}}}$$

where *v*_sink_ is the total value (cost, if negative) of seaweed farmed for CDR in $ tDW^−1^, *v*_cprice_ is a theoretical carbon price, *c*_swtsink_ is as calculated in equation (11), *c*_eqtsink_ is as calculated in equation (12) and *s*_dw_ is the annually harvested seaweed DW in t km^−2^. We did not assume any carbon price in our Monte Carlo simulations (*v*_cprice_ is equal to zero), making *v*_sink_ negative and thus representing a net cost.

To calculate net carbon impacts, our model included uncertainty in the efficiency of using the growth and subsequent deep-sea deposition of seaweed as a CDR method. The uncertainty is expected to include the effects of reduced phytoplankton growth from nutrient competition, the relationship between air–sea gas exchange and overturning circulation (hereafter collectively referred to as the ‘atmospheric removal fraction’) and the fraction of deposited seaweed carbon that remains sequestered for at least 100 yr. The total amount of atmospheric CO_2_ removed by sinking seaweed was calculated as:

14 $$e_{seqsink} = k_{atm} \times k_{fseq} \times \frac{{tC}}{{tDW}} \times \frac{{tCO_2}}{{tC}}$$

where *e*_seqsink_ is net atmospheric CO_2_ sequestered annually in t km^−2^, *k*_atm_ is the atmospheric removal fraction and *k*_fseq_ is the spatially explicit fraction of sunk seaweed carbon that remains sequestered for at least 100 yr (see ref. 57).

The emissions from transporting harvested seaweed to the optimal sinking location were calculated as:

15 $$e_{swtsink} = e_{transbase} \times d_{sink} \times s_{ww}$$

where *e*_swtsink_ is the total annual CO_2_ emissions from transporting harvested seaweed to the optimal sinking location in tCO_2_ km^−2^, *e*_transbase_ is the CO_2_ emissions (tons) from transporting 1 ton of material 1 km on a barge (tCO_2_ per t-km), *d*_sink_ is the distance in km to the economically optimized sinking location and *s*_ww_ is the annually harvested seaweed wet weight in t km^−2^ as in equation (10). Since the unit for *e*_transbase_ is tCO_2_ per t-km, the emissions from transporting seaweed to the optimal sinking location are equal to $e_{\mathrm{transbase}} \times d_{\mathrm{sink}} \times s_{\mathrm{ww}}$, and the emissions from transporting seaweed from the optimal sinking location back to the farm are equal to 0 (since the seaweed has already been deposited, the seaweed mass to transport is now 0). Note that this does not yet include transport emissions from transport of equipment post-seaweed-deposition (see equation 16 below and Supplementary Fig. 16).

The emissions associated with transporting replacement equipment (for example, lines, buoys, anchors) to the farming location and hauling back used equipment at the end of its assumed lifetime (1 yr for seeded line, 5–20 yr for capital equipment by equipment type)^28,41^ in the sinking CDR pathway were calculated as:

16 $$e_{eqtsink} = \left( {e_{transbase} \times \left( {2 \times d_{sink}} \right) \times m_{eq}} \right) + (e_{transbase} \times d_{port} \times m_{eq})$$

where *e*_eqtsink_ is the total annualized CO_2_ emissions in tons from transporting both used and replacement equipment, *e*_transbase_ is the CO_2_ emissions from transporting 1 ton of material 1 km on a barge, *m*_eq_ is the annualized equipment mass in tons, *d*_sink_ is the distance in km to the economically optimized sinking location and *d*_port_ is the ocean distance to the nearest port in km. We assumed that the harvesting barge travels from the farming location directly to the optimal sinking location with harvested seaweed and replaced (used) equipment in tow (including used seeded line and annualized mass of used capital equipment), sinks the harvested seaweed, returns to the farm location and then returns to the nearest port. These calculations assumed the shortest sea-route distance (see Distance to optimal sinking point).

Net CO_2_ emissions removed from the atmosphere by sinking seaweed were thus calculated as:

17 $$e_{remsink} = \frac{{\left( {e_{seqsink} - \left( {e_{swtsink} + e_{eqtsink}} \right)} \right)}}{{s_{dw}}}$$

where *e*_remsink_ is the net atmospheric CO_2_ removed per ton of seaweed DW, *e*_seqsink_ is as calculated in equation (14), *e*_swtsink_ is as calculated in equation (15), *e*_eqtsink_ is as calculated in equation (16) and *s*_dw_ is the annually harvested seaweed DW in t km^−2^.

### Net cost of climate benefits

#### Sinking

To calculate the total net cost and emissions from the production, harvesting and transport of seaweed for CDR, we combined the cost and emissions from the sinking-pathway cost and value modules. The total net cost of seaweed CDR per DW ton of seaweed was calculated as:

18 $$c_{sinknet} = c_{prod} - v_{sink}$$

where *c*_sinknet_ is the total net cost of seaweed for CDR per DW ton harvested, *c*_prod_ is the net production cost per DW ton as calculated in equation (6) and *v*_sink_ is the net value (or cost, if negative) per ton seaweed DW as calculated in equation (13).

The total net CO_2_ emissions removed per DW ton of seaweed were calculated as:

19 $$e_{sinknet} = e_{remsink} - e_{prod}$$

where *e*_sinknet_ is the total net atmospheric CO_2_ removed per DW ton of seaweed harvested annually (tCO_2_ tDW^−1^ yr^−1^), *e*_remsink_ is the net atmospheric CO_2_ removed via seaweed sinking annually as calculated in equation (17) and *e*_prod_ is the net CO_2_ emitted from production and harvesting of seaweed annually as calculated in equation (9). For each Monte Carlo simulation, locations where *e*_sinknet_ is negative (that is, net emissions rather than net removal) were not included in subsequent calculations since they would not be contributing to CDR in that location under the given scenario. Note that these net emissions cases only occur in areas far from port in specific high-emissions scenarios. Even in such cases, most areas still contribute to CO_2_ removal (negative emissions), hence costs from locations with net removal were included.

Total net cost was then divided by total net emissions to get a final value for cost per ton of atmospheric CO_2_ removed:

20 $$c_{pertonsink} = \frac{{c_{sinknet}}}{{e_{sinknet}}}$$

where *c*_pertonsink_ is the total net cost per ton of atmospheric CO_2_ removed via seaweed sinking ($ per tCO_2_ removed), *c*_sinknet_ is total net cost per ton seaweed DW harvested as calculated in equation (18) ($ tDW^−1^) and *e*_sinknet_ is the total net atmospheric CO_2_ removed per ton seaweed DW harvested as calculated in equation (19) (tCO_2_ tDW^−1^).

#### GHG emissions mitigation

Instead of sinking seaweed for CDR, seaweed can be used to make products (including but not limited to food, animal feed and biofuels). Replacing convention products with seaweed-based products can result in ‘avoided emissions’ if the emissions from growing, harvesting, transporting and converting seaweed into products are less than the total greenhouse gas emissions (including non-CO_2_ GHGs) embodied in conventional products that seaweed-based products replace.

When seaweed is used to make products, we assumed it is transported back to the nearest port immediately after being harvested. The annualized cost to transport the harvested seaweed and replacement equipment (for example, lines, buoys, anchors) was calculated as:

21 $$c_{transprod} = \frac{{\left( {c_{transbase} \times d_{port} \times \left( {s_{ww} + m_{eq}} \right)} \right)}}{{s_{dw}}}$$

where *c*_transprod_ is the annualized cost per ton seaweed DW to transport seaweed and equipment back to port from the farm location, *c*_transbase_ is the cost to transport 1 ton of material 1 km on a barge, *m*_eq_ is the annualized equipment mass in tons, *d*_port_ is the ocean distance to the nearest port in km, *s*_ww_ is the annual wet weight of seaweed harvested per km^2^ as calculated in equation (10) and *s*_dw_ is the annual DW of seaweed harvested per km^2^.

The total value of seaweed that is used for seaweed-based products was calculated as:

22 $$v_{product} = v_{mkt} - \left( {c_{transprod} + c_{conv}} \right)$$

where *v*_product_ is the total value (cost, if negative) of seaweed used for products ($ tDW^−1^), *v*_mkt_ is how much each ton of seaweed would sell for, given the current market price of conventional products that seaweed-based products replace ($ tDW^−1^), *c*_transprod_ is as calculated in equation (21) and *c*_conv_ is the cost to convert each ton of seaweed to a usable product ($ tDW^−1^).

The annualized CO_2_ emissions from transporting harvested seaweed and equipment back to port were calculated as:

23 $$e_{transprod} = \frac{{\left( {e_{transbase} \times d_{port} \times \left( {s_{ww} + m_{eq}} \right)} \right)}}{{s_{dw}}}$$

where *e*_transprod_ is the annualized CO_2_ emissions per ton seaweed DW to transport seaweed and equipment back to port from the farm location, *e*_transbase_ is the CO_2_ emissions from transporting 1 ton of material 1 km on a barge, *m*_eq_ is the annualized equipment mass in tons, *d*_port_ is the ocean distance to the nearest port in km, *s*_ww_ is the annual wet weight of seaweed harvested per km^2^ as calculated in equation (10) and *s*_dw_ is the annual DW of seaweed harvested per km^2^.

Total emissions avoided by each ton of harvested seaweed DW were calculated as:

24 $$e_{avprod} = e_{subprod} - \left( {e_{transprod} + e_{conv}} \right)$$

where *e*_avprod_ is total CO_2_-eq emissions avoided per ton of seaweed DW per year (including non-CO_2_ GHGs using a GWP time period of 100 yr), *e*_subprod_ is the annual CO_2_-eq emissions avoided per ton seaweed DW by replacing a conventional product with a seaweed-based product, *e*_transprod_ is as calculated in equation (23) and *e*_conv_ is the annual CO_2_ emissions per ton seaweed DW from converting seaweed into usable products. *e*_subprod_ was calculated by converting seaweed DW to caloric content^58^ for food/feed and comparing emissions intensity per kcal to agricultural products^24^, or by converting seaweed DW into equivalent biofuel content with a yield of 0.25 tons biofuel per ton DW^59^ and dividing the CO_2_ emissions per ton fossil fuel by the seaweed biofuel yield.

To calculate the total net cost and emissions from the production, harvesting, transport and conversion of seaweed for products, we combined the cost and emissions from the product-pathway cost and value modules. The total net cost of seaweed for products per ton DW was calculated as:

25 $$c_{prodnet} = c_{prod} - v_{product}$$

where *c*_prodnet_ is the total net cost per ton DW of seaweed harvested for use in products, *c*_prod_ is the net production cost per ton DW as calculated in equation (6) and *v*_product_ is the net value (or cost, if negative) per ton DW as calculated in equation (22).

The total net CO_2_-eq emissions avoided per ton DW of seaweed used in products were calculated as:

26 $$e_{prodnet} = e_{avprod} - e_{prod}$$

where *e*_prodnet_ is the total net CO_2_-eq emissions avoided per ton DW of seaweed harvested annually (tCO_2_ tDW^−1^ yr^−1^), *e*_avprod_ is the net CO_2_-eq emissions avoided by seaweed products annually as calculated in equation (24) and *e*_prod_ is the net CO_2_ emitted from production and harvesting of seaweed annually as calculated in equation (9). For each Monte Carlo simulation, locations where *e*_prodnet_ is negative (that is, net emissions rather than net emissions avoided) were not included in subsequent calculations since they would not be avoiding any emissions in that scenario.

Total net cost was then divided by total net emissions avoided to get a final value for cost per ton of CO_2_-eq emissions avoided:

27 $$c_{pertonprod} = \frac{{c_{prodnet}}}{{e_{prodnet}}}$$

where *c*_pertonprod_ is the total net cost per ton of CO_2_-eq emissions avoided by seaweed products ($ per tCO_2_-eq avoided), *c*_prodnet_ is total net cost per ton seaweed DW harvested for products as calculated in equation (25) ($ tDW^−1^) and *e*_prodnet_ is total net CO_2_-eq emissions avoided per ton seaweed DW harvested for products as calculated in equation (26) (tCO_2_ tDW^−1^).

### Parameter ranges for Monte Carlo simulations

For technoeconomic parameters with two or more literature values (see Supplementary Table 1), we assumed that the maximum literature value reflected the 95th percentile and the minimum literature value represented the 5th percentile of potential costs or emissions. For parameters with only one literature value, we added ±50% to the literature value to represent greater uncertainty within the modelled parameter range. Values at each end of parameter ranges were then rounded before Monte Carlo simulations as follows: capital costs, operating costs and harvest costs to the nearest $10,000 km^−2^, labour costs and insurance costs to the nearest $1,000 km^−2^, line costs to the nearest $0.05 m^−1^, transport costs to the nearest $0.05 t^−1^ km^−1^, transport emissions to the nearest 0.000005 tCO_2_ t^−1^ km^−1^, maintenance transport emissions to the nearest 0.0005 tCO_2_ km^−1^, product-avoided emissions to the nearest 0.1 tCO_2_-eq tDW^−1^, conversion cost down to the nearest $10 tDW^−1^ on the low end of the range and up to the nearest $10 tDW^−1^ on the high end of the range, and conversion emissions to the nearest 0.01 tCO_2_ tDW^−1^.

We extended the minimum range values of capital costs to $10,000 km^−2^ and transport emissions to 0 to reflect potential future innovations, such as autonomous floating farm setups that would lower capital costs and net-zero emissions boats that would result in 0 transport emissions. To calculate the minimum value of $10,000 km^−2^ for a potential autonomous floating farm, we assumed that the bulk of capital costs for such a system would be from structural lines and flotation devices, and we therefore used the annualized structural line (system rope) and buoy costs from ref. 41 rounded down to the nearest $5,000 km^−2^. The full ranges used for our Monte Carlo simulations and associated literature values are shown in Supplementary Table 1.

### Distance to optimal sinking point

Distance to the optimal sinking point was calculated using a weighted distance transform (path-finding algorithm, modified from code in ref. 60) that finds the shortest ocean distance from each seaweed growth pixel to the location at which the net CO_2_ removed is maximized (including impacts of both increased sequestration fraction and transport emissions for different potential sinking locations) and the net cost is minimized. This is not necessarily the location in which the seaweed was grown, since the fraction of sunk carbon that remains sequestered for 100 yr is spatially heterogeneous (see ref. 57). For each ocean grid cell, we determined the cost-optimal sinking point by iteratively calculating equations (11–20) and assigning *d*_sink_ as the distance calculated by weighted distance transform to each potential sequestration fraction (0.01–1.00) in increments of 0.01. Except for transport emissions, the economic parameter values used for these calculations were the averages of unrounded literature value ranges; we assumed that the maximum literature value reflected the 95th percentile and the minimum literature value represented the 5th percentile of potential costs or emissions, or for parameters with only one literature value, we added ±50% to the literature value to represent greater uncertainty within the modelled parameter range. For transport and maintenance transport emissions, we extended the minimum values of the literature ranges to zero to reflect potential net-zero emissions transport options and used the mean values of the resulting ranges. The *d*_sink_ that resulted in minimum net cost per ton CO_2_ for each ocean grid cell was saved as the final *d*_sink_ map, and the associated sequestration fraction value that the seaweed is transported to via *d*_sink_ was assigned to the original cell where the seaweed was farmed and harvested (Supplementary Fig. 19). If the cost-optimal location to sink using this method was the same cell where the seaweed was harvested, then *d*_sink_ was 0 km and the sequestration fraction was not modified from its original value (Supplementary Fig. 18).

### Comparison of gigaton-scale sequestration area to previous estimates

Previous related work estimating the ocean area suitable for macroalgae cultivation^13^ and/or the area that might be required to reach gigaton-scale carbon removal via macroalgae cultivation^13,19,36^ has yielded a wide range of results, primarily due to differences in modelling methods. For example, Gao et al. (2022)^36^ estimate that 1.15 million km^2^ would be required to sequester 1 GtCO_2_ annually when considering carbon lost from seaweed biomass/sequestered as particulate organic carbon (POC) and refractory dissolved organic carbon (rDOC), and assume that the harvested seaweed is sold as food such that the carbon in the harvested seaweed is not sequestered. The area (0.4 million km^2^) required to sequester 1 GtCO_2_ in our study assumes that all harvested seaweed is sunk to the deep ocean to sequester carbon.

Additionally, Wu et al.^19^ estimates that roughly 12 GtCO_2_ could be sequestered annually via macroalgae cultivation in approximately 20% of the world ocean area (that is, 1.67% ocean area per GtCO_2_), which is a much larger area per GtCO_2_ than our estimate of 0.110% ocean area. This notable difference arises for several reasons (including differences in yields, which in Wu et al. are around 500 tDW yr^−1^ in the highest-yield areas, whereas yields in our cheapest sequestration areas from G-MACMODS average 3,400 tDW km^−2^ yr^−1^) that arise from differences in model methodology. First, Wu et al. model temperate brown seaweeds, while our study considers different seaweed types, many of which have higher growth rates, and uses the most productive seaweed type for each ocean grid cell. The G-MACMODS seaweed growth model we use also has a highly optimized harvest schedule, includes luxury nutrient uptake (a key feature of macroalgal nutrient physiology) and does not directly model competition with phytoplankton during seaweed growth. Finally, tropical red seaweeds (the seaweed type in our cheapest sequestration areas) grow year-round, while others, such as the temperate brown seaweeds modelled by Wu et al., only grow seasonally. These differences all contribute to higher productivity in our model, leading to a smaller area required for gigaton-scale CO_2_ sequestration compared with Wu et al.

Conversely, the ocean areas we model for seaweed-based CO_2_ sequestration or GHG emissions avoided are much larger than the 48 million km^2^ that Froehlich et al.^13^ estimate to be suitable for macroalgae farming globally. Although our maps show productivity and costs everywhere, the purpose of our modelling was to evaluate where different types of seaweed grow best and how production costs and product values vary over space, to highlight the lowest-cost areas (which are often the highest-producing areas) under various technoeconomic assumptions.

### Comparison of seaweed production costs to previous estimates

Although there are not many estimates of seaweed production costs in the scientific literature, our estimates for the lowest-cost 1% area of the ocean ($190–$2,790 tDW^−1^) are broadly consistent with previously published results: seaweed production costs reported in the literature range from $120 to $1,710 tDW^−1^ (refs. 40, 41, 61, 62), but are highly dependent on assumed seaweed yields. For example, Camus et al.^41^ calculate a cost of $870 tDW^−1^ assuming a minimum yield of 12.4 kgDW m^−1^ of cultivation line (equivalent to 8.3 kgDW m^−2^ using 1.5 m spacing between lines). Using the economic values from Camus et al. but with our estimates of average yield for the cheapest 1% production cost areas (2.6 kgDW m^−2^) gives a much higher average cost of $2,730 tDW^−1^. Contrarily, van den Burg et al.^40^ calculate a cost of $1,710 tDW^−1^ using a yield of 20 tDW ha^−1^ (that is, 2.0 kg m^−2^). Instead assuming the average yield to be that from our lowest-cost areas (that is, 2.6 kgDW m^−2^ or 26 tDW ha^−1^) would decrease the cost estimated by van den Burg et al. (2016) to $1,290 tDW^−1^. Most recently, Capron et al.^62^ calculate an optimistic scenario cost of $120 tDW^−1^ on the basis of an estimated yield of 120 tDW ha^−1^ (12 kg m^−2^; over 4.5 times higher than the average yield in our lowest-cost areas). Again, instead assuming the average yield to be that in our lowest-cost areas would raise Capron et al.’s production cost to $540 tDW^−1^ (between the $190–$880 tDW^−1^ minimum to median production costs in the cheapest 1% areas from our model; Fig. 1a,b).

### Data sources

#### Seaweed biomass harvested

We used spatially explicit data for seaweed harvested globally under both ambient and limited-nutrient scenarios from the G-MACMODS seaweed growth model^33^.

#### Fraction of deposited carbon sequestered for 100 yr

We used data from ref. 57 interpolated to our 1/12-degree grid resolution.

#### Distance to the nearest port

We used the Distance from Port V1 dataset from Global Fishing Watch (https://globalfishingwatch.org/data-download/datasets/public-distance-from-port-v1) interpolated to our 1/12-degree grid resolution.

#### Significant wave height

We used data for annually averaged significant wave height from the European Center for Medium-range Weather Forecasts (ECMWF) interpolated to our 1/12-degree grid resolution.

#### Ocean depth

We used data from the General Bathymetric Chart of the Oceans (GEBCO).

#### Shipping lanes

We used data of Automatic Identification System (AIS) signal count per ocean grid cell, interpolated to our 1/12-degree grid resolution. We defined a major shipping lane grid cell as any cell with >2.25 × 10^8^ AIS signals, a threshold that encompasses most major trans-Pacific and trans-Atlantic shipping lanes as well as major shipping lanes in the Indian Ocean, the North Sea, and coastal routes worldwide.

#### Marine protected areas (MPAs)

We used data from the World Database on Protected Areas (WDPA) and defined an MPA as any ocean MPA >20 km^2^.

### Reporting summary

Further information on research design is available in the Nature Portfolio Reporting Summary linked to this article.

## Supplementary information

Supplementary Information Supplementary Figs. 1–20 and Tables 1 and 2.

Reporting Summary

## Data Availability

Data from this study are publicly available through Dryad at 10.7280/D13H59.

## Code availability

Code was run using Python 3.9 and is publicly available through Zenodo at 10.5281/zenodo.7262015.
