# Author Correction: Economic and biophysical limits to seaweed farming for climate change mitigation

**DOI:** 10.1038/s41477-023-01393-1

**Published:** 2023-03-14

**Authors:** Julianne DeAngelo, Benjamin T. Saenz, Isabella B. Arzeno-Soltero, Christina A. Frieder, Matthew C. Long, Joseph Hamman, Kristen A. Davis, Steven J. Davis

**Affiliations:** 1 Department of Earth System Science, University of California, Irvine, Irvine, CA USA; 2 Biota.earth, Berkeley, CA USA; 3 Department of Civil and Environmental Engineering, Stanford University, Stanford, CA USA; 4 Southern California Coastal Water Research Project, Costa Mesa, CA USA; 5 National Center for Atmospheric Research, Boulder, CO USA; 6 Earthmover, New York, NY USA; 7 Department of Civil and Environmental Engineering, University of California, Irvine, Irvine, CA USA

**Keywords:** Climate-change mitigation, Environmental economics, Climate-change mitigation

Correction to: *Nature Plants* 10.1038/s41477-022-01305-9. Published online 23 December 2022.

In the version of this article initially published, in the Results subsection “Costs and benefits of large-scale seaweed farming”, the percentages of ocean area farmed to reach 1 Gt and 3 Gt of CO_2_ sequestered by sinking seaweed in the ambient nutrient scenario were incorrect due to typographical errors. As a result, in the sentence beginning “In the optimistic case”, “0.85%” is now “0.110%”, “310,000 km^2^” is now “400,000 km^2^”, and “Poland” now reads as “Zimbabwe”. In the sentence following, “is slightly higher: 0.036% and 0.099%” now reads as “is 0.035% and 0.100%” and “$40” is now “$30”. Furthermore, in the penultimate sentence of this paragraph, “ocean areas of 0.09–0.10% and 0.28–0.37%” now reads as “ocean areas of 0.085–0.100% and 0.285–0.410%” and “roughly 320,000–360,000 km^2^ and 1,010,000–1,330,000 km^2^” is now “roughly 310,000–360,000 km^2^ and 1,030,000–1,480,000 km^2^”. Calculated values in this subsection have been revised to reflect these corrections: in the sentence beginning “Average costs at the median of Monte Carlo”, “$1,110–$2,100” is now “$1,120–$2,090”; and in the paragraph beginning “Despite being a small percentage”, “17%” is now “18%” and “61%” is now “64%”. Corresponding values in the Methods subsection “Comparison of gigaton-scale sequestration area to previous estimates” and in Supplementary Figures 10 and 11 have been updated accordingly. The errors have been corrected in the HTML and PDF versions of the article.

