# Supplementary material for: Economic and biophysical limits to seaweed farming for climate change mitigation
Source: Nat Plants. 2022 Dec 23;9(1):45–57. doi: 10.1038/s41477-022-01305-9 (PMC9873559; doi:10.1038/s41477-022-01305-9)
Supplement: Supplementary file 1 — Supplementary Figs. 1–20 and Tables 1 and 2. [file 41477_2022_1305_MOESM1_ESM.pdf]

nature plants

Article https://doi.org/10.1038/s41477-022-01305-9

Economic and biophysical limits to seaweed farming for climate change mitigation

In the format provided by the authors and unedited

Supplementary Information

Economic and biophysical limits to seaweed farming for climate change mitigation

DeAngelo et al.

Please note that references for supplementary figures and tables are listed at the end of the Supplementary Information.

Supplementary Table 1 | Technoeconomic model variables.

Variable	Unit	Model Range	Values reported in literature
Capital costs	$/km^2/year$	10,000 – 1,000,000	929,676 [1]<br>550,000 – 950,000 [2]<br>375,910 [3]<br>210,580 [4]
Operating and maintenance costs	$/km^2/year$	60,000 – 70,000	69,000 [1]<br>63,320 [4]
Seeded line cost (includes hatchery costs)	$/m	0.05 – 1.45	1.38 [1]<br>0.13 [4]
Labor costs (excludes harvest labor)	$/km^2/year$	38,000 – 120,000	115,485 [4]<br>41,800 [1]
Harvest costs (includes harvest labor, excludes harvest transport)	$/km^2/harvest$	120,000 – 400,000	381,265 [4]<br>138,000 [1]
Transport cost per ton of material (includes loading/unloading costs)	$/t/km	0.1 – 0.35	0.225 [1]
Transport emissions per ton of material	$tCO_2/t/km$	0 – 0.000045	0.00003 [5]
Maintenance boat emissions	tCO_2/km	0 – 0.0035	0.0023653 (calculated using methods from [5,6])
Insurance costs	$/km^2/year$	35,000 – 105,000	70,000 [1]
Aquaculture license costs	$/km^2/year$	1,000 – 2,000	1,420 [4]
Atmospheric removal fraction	fraction (unitless)	0.4 – 1	0.4 – 0.75 [7]<br>0.5 (global average, from preliminary experiment by authors using [8] informed by [9])
Seaweed yield	tDW/km^2/year	5th, 25th, median, 75th, and 95th percentile maps (randomly selected each simulation from normal distribution)	Results from G-MACMODS biophysical growth model Monte Carlo analysis [10]

Seaweed market value for product end-use	$/tDW	400 – 800	Food: 500-800 (dried seaweed wholesale price from [11])<br><br>Feed: 400-500 (values per ton dry animal feed and soybean meal from [1,12], assuming a direct replacement with dry seaweed)<br><br>Fuel: 430 (dried seaweed price for bioethanol production, calculated based on bioethanol yield per ton seaweed (0.25) and average of 2021-2022 historical E85 fuel prices ($3.76/GGE) from [13], modeled range 400-500)<br><br>Not product-specific: 400 (dried seaweed market price of $400/tDW from [14])
Conversion cost	$/tDW	20 – 80	48 (calculated with data from [15] assuming plant meets full feedstock capacity)
Conversion emissions	tCO_2/tDW	0 – 0.01	0.0057 (calculated using data and methods from [15])
Depth impact on capex	multiplier (unitless)	0 – 1	1 (estimation used in [1] that offshore depth can double capital costs)
Significant wave height impact on capex	multiplier (unitless)	0 – 1	Author assumption that high waviness impacts capital lifetime similarly to depth impact
GHG emissions avoided by replacement with seaweed product	tCO_2e/tDW	0.7 – 6.0	Food: 1-6 (considering global average emissions from GHGs per kcal for pulses, vegetables, fruits, oil crops, and cereals, from [16])<br><br>Feed: 1-3.1 (considering global average emissions from GHGs per kcal for oil crops and cereals, +- 50% uncertainty, from [16])<br><br>Fuel: 0.7-1 (assuming 3.2-3.5 tCO_2/t fossil fuel by fuel type from [17], 0.25t bioethanol/tDW from [15], and energy density equivalence conversions by fuel type)

Supplementary Table 2 | Constants in model.

Parameter	Unit	Model Range	Source
Total length of cultivation line per unit area	m/km^2	Tropical red: 5,000,000<br>Temperate red: 20,000,000<br>Tropical brown: 751,880<br>Temperate brown: 666,667	Calculated using species-specific line spacing from [10]
Capital and other annualized equipment mass	t/km^2/year	Tropical red: 1,231.87<br>Temperate red: 4,927.50<br>Tropical brown: 185.24<br>Temperate brown: 164.25	Calculated using line spacing (above) and methods from extended methods in [5]
Number of maintenance trips	trips/km^2/year	6	[5]
Fraction of sunk carbon sequestered for 100 years	fraction (unitless)	0 – 1	[18]
Depth beyond which capex increases via depth_mult	m	500	[19]
Significant wave height beyond which capex increases via wave_mult	m	3	[20]
Seaweed carbon fraction	tC/tDW	0.3	[10,21]
Seaweed caloric content	Kcal/tDW	2,980,000	[22]
Bioethanol yield from seaweed	t/tDW	0.25	[15]

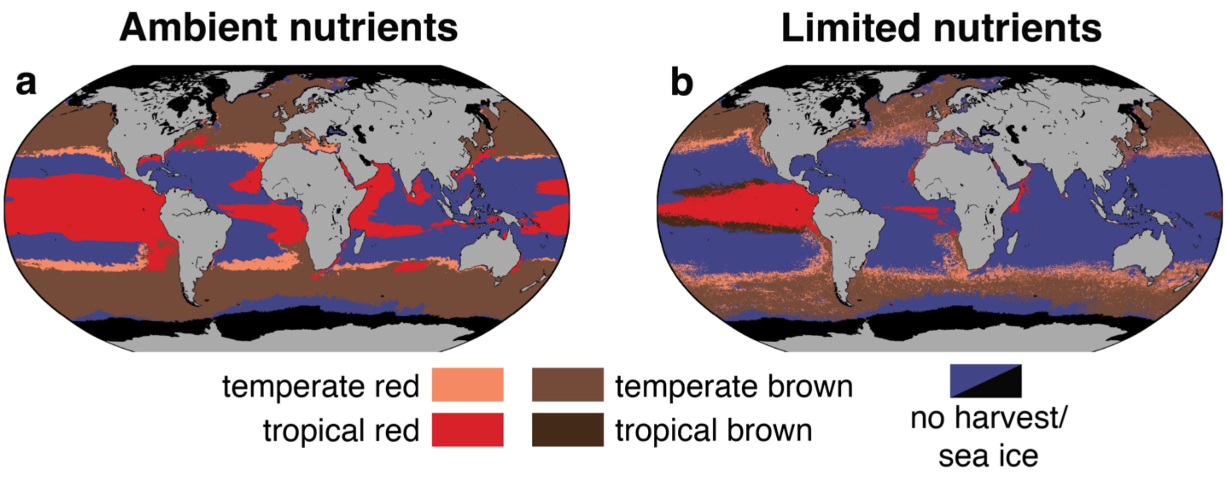

Supplementary Figure 1 | Preferred seaweed type. The seaweed type that results in the most harvested biomass is shown for each ocean grid cell for the G-MACMODS standard run ambient nutrient scenario (**a**) and limited nutrient scenario (**b**). Modified from [10].

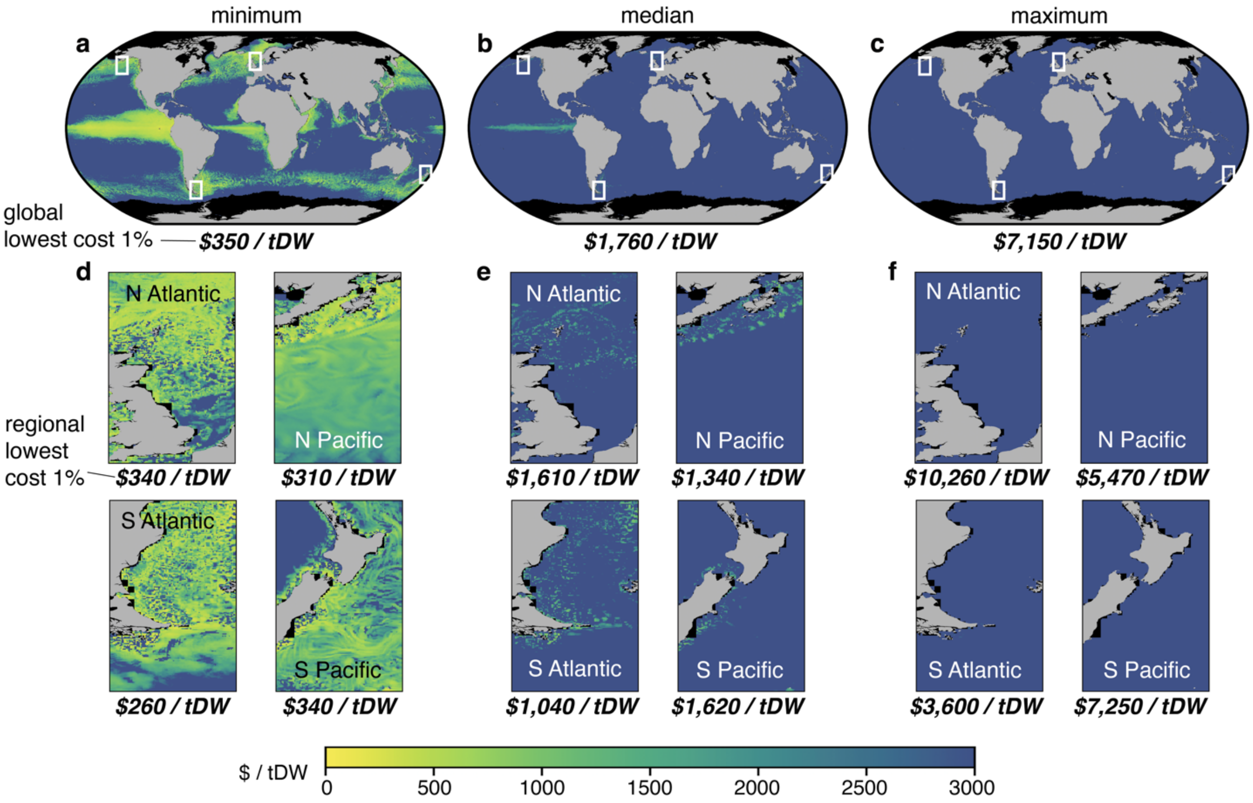

Supplementary Figure 2 | Seaweed production and harvest cost using limited nutrient scenario. Estimated seaweed production costs vary considerably depending on assumed costs of farming capital, seeded lines, labor, and harvest (transport of harvested seaweed is not included). Across limited nutrient simulations, average farming cost in the 1% of global ocean areas with lowest cost ranges from $350/tDW (**a**) to $7,150/tDW (**c**), with a median of $1,760/tDW (**b**). Regional insets (**d-f**) reveal small-scale features in particularly low-cost areas.

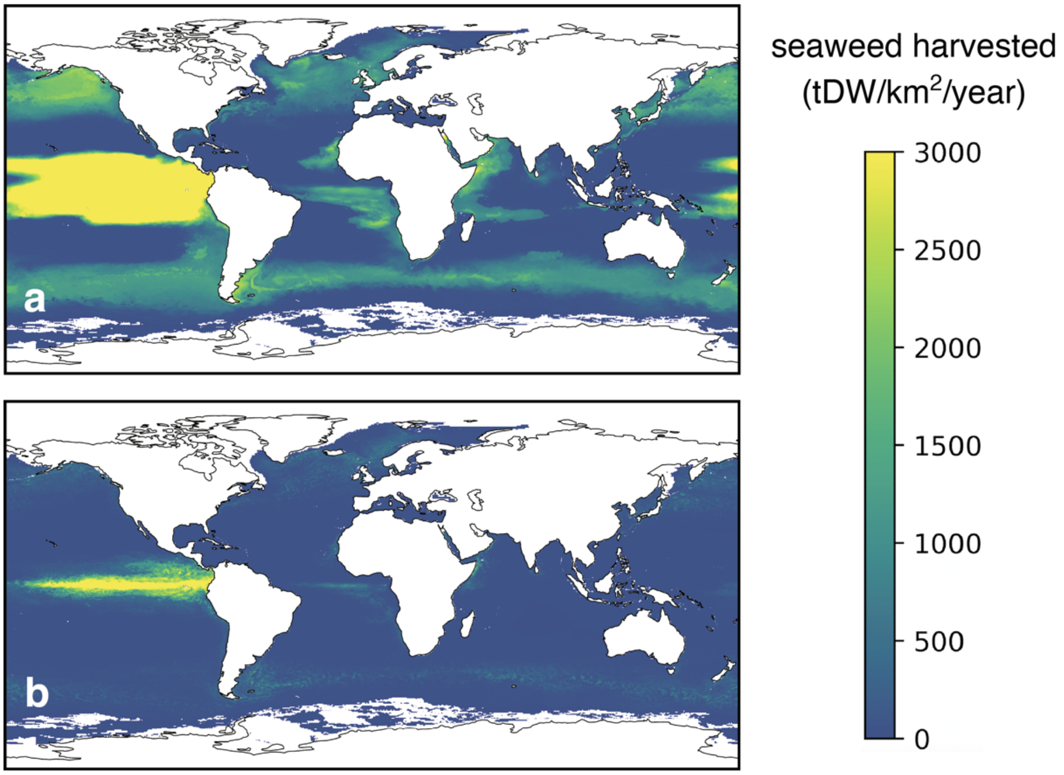

Supplementary Figure 3 | Annual seaweed biomass harvested. Maps show amount of seaweed harvested annually (tDW/km^2/year) from median Monte Carlo results using ambient nutrients (**a**) and limited nutrients (**b**) in G-MACMODS [10].

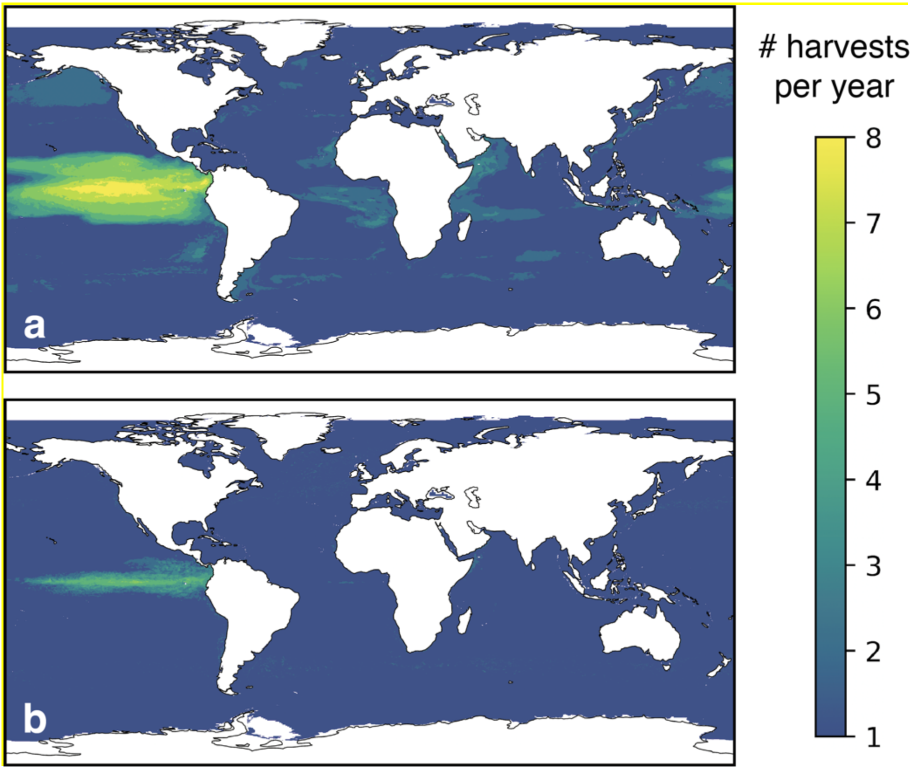

Supplementary Figure 4 | Number of harvests required to reach maximum annual yield. Maps show number of harvests per year from median Monte Carlo results using ambient nutrients (**a**) and limited nutrients (**b**) in G-MACMODS [10].

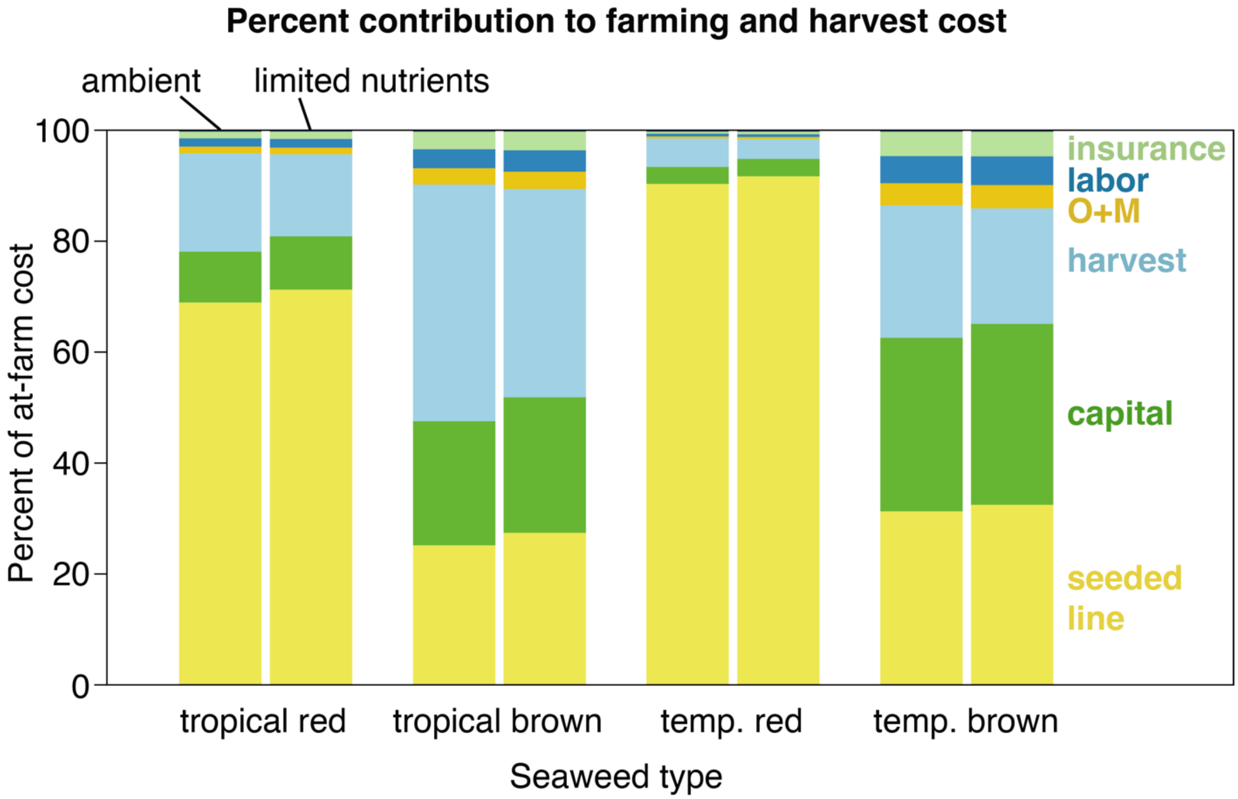

Supplementary Figure 5 | Summary of total farming cost breakdown per km². Percent of total $/km² seaweed farming cost for four seaweed types in ambient nutrients (left bar for each type) and limited nutrients (right bar for each type) simulations. Note: does not include transportation costs.

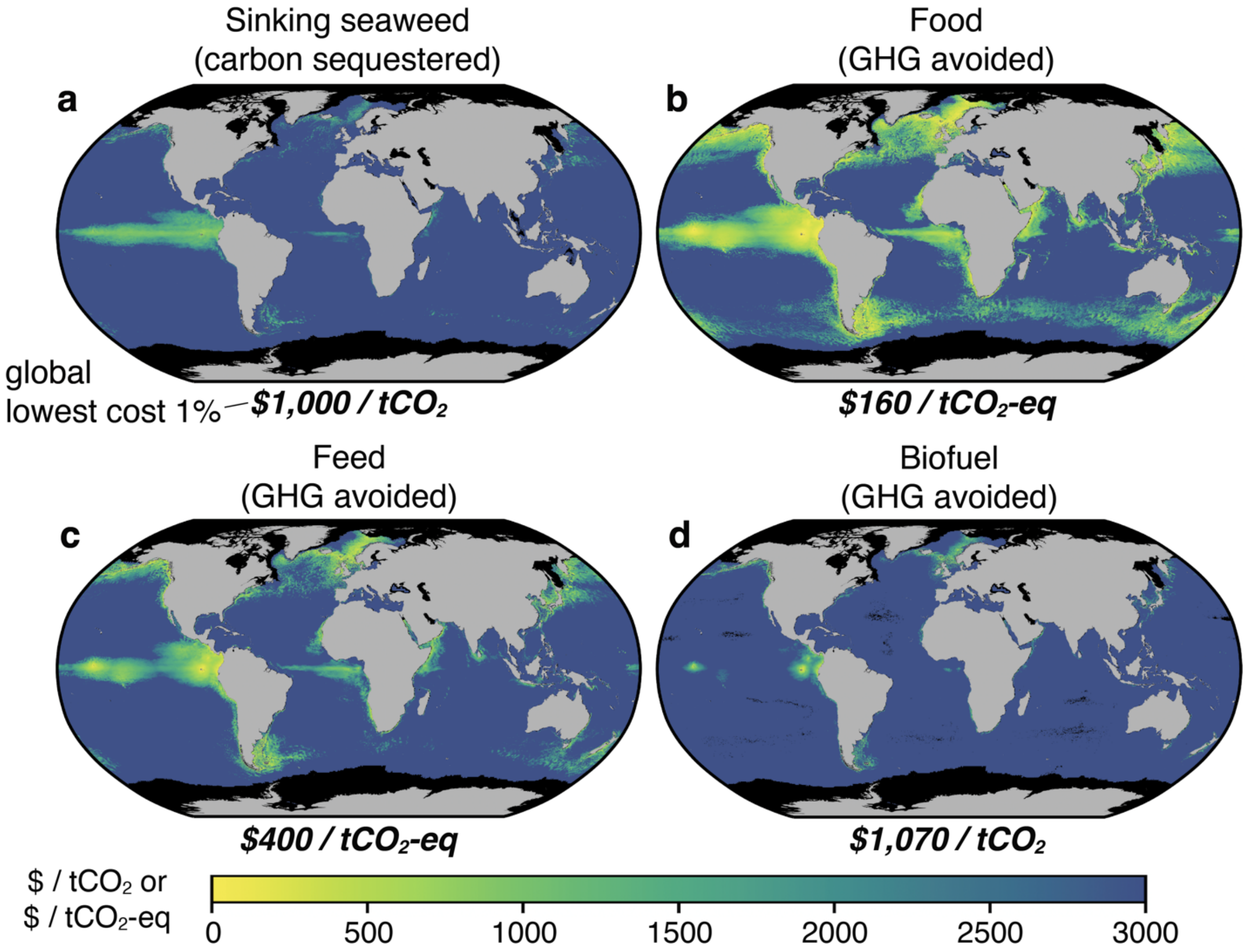

Supplementary Figure 6 | Net cost of potential seaweed climate benefits for limited nutrient simulations. Costs of using farmed seaweed to sequester carbon or avoid GHG emissions vary in space according to estimated production costs as well as spatially-explicit differences in the costs and net emissions of transportation, sinking or conversion, and replacement of conventional market alternatives with seaweed products. Differentiation between seaweed product groups (**b-d**) is based on emissions avoided by seaweed products and market value for each product type. Maps show costs when propagating the most optimistic assumptions (5th percentile costs) from limited nutrient simulations. Average cost in the 1% of global ocean areas with lowest cost ranges from \$160/tCO_2-eq avoided when seaweed is used for food (**b**) to \$1,070/tCO_2 avoided when seaweed is used to produce biofuel (**d**).

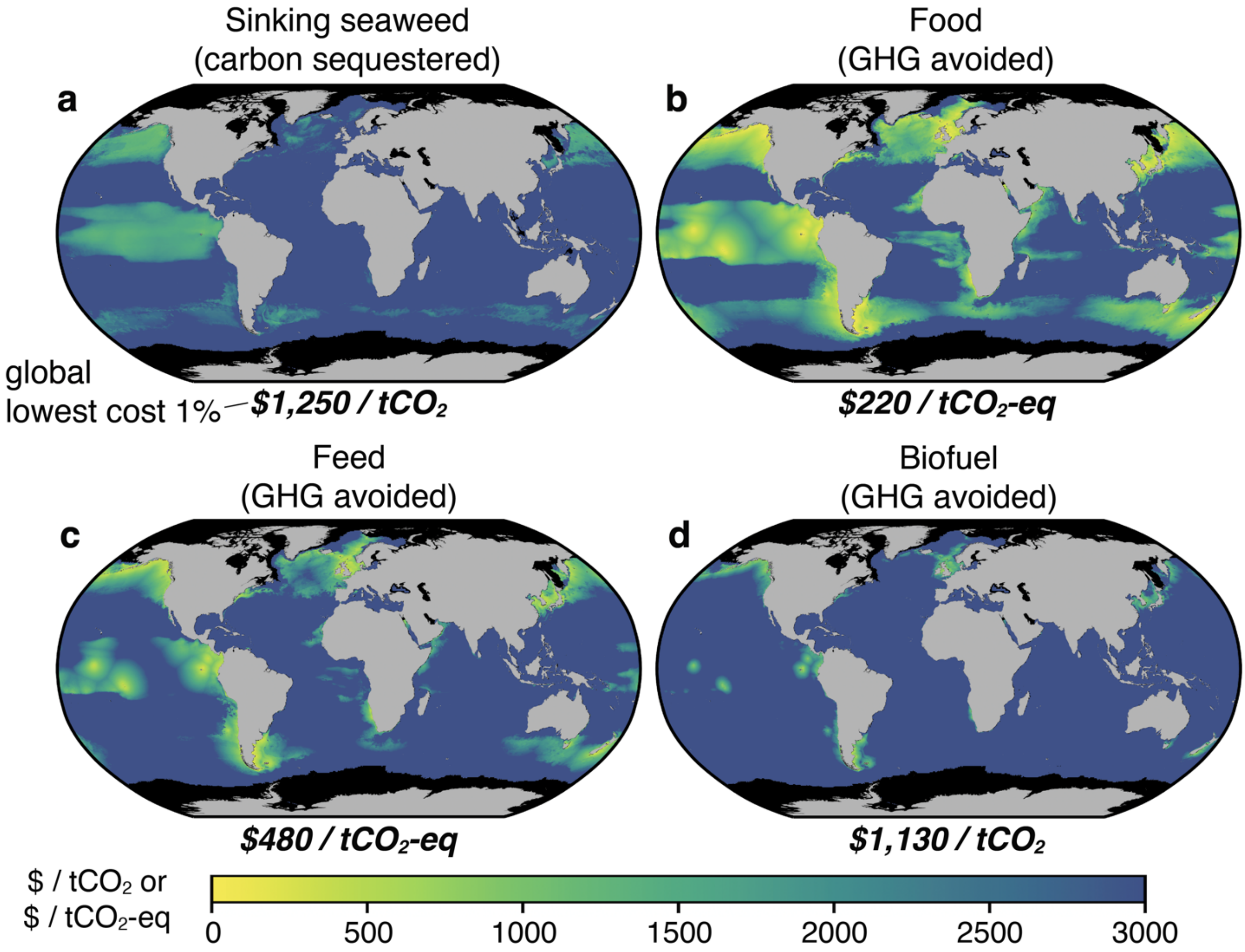

Supplementary Figure 7 | Median net cost of potential seaweed climate benefits. Costs of using farmed seaweed to sequester carbon or avoid GHG emissions vary in space according to estimated production costs as well as spatially-explicit differences in the costs and net emissions of transportation, sinking or conversion, and replacement of conventional market alternatives with seaweed products. Differentiation between seaweed product groups (**b-d**) is based on emissions avoided by seaweed products and market value for each product type. Maps show median costs from ambient nutrient simulations. Average cost in the 1% of global ocean areas with lowest cost ranges from \$220/tCO_2-eq avoided when seaweed is used for food (**b**) to \$1,250/tCO_2 sequestered by sinking seaweed (**a**).

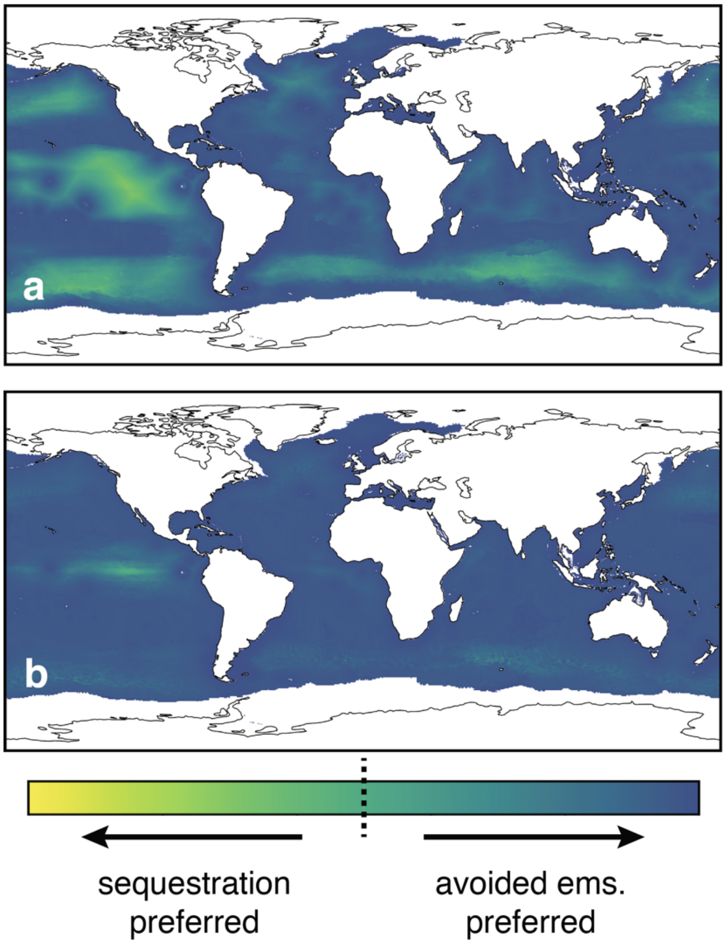

Supplementary Figure 8 | Economic preference for sinking or products by location. Maps show the average across all ambient nutrient (**a**) and limited nutrient (**b**) simulations of whether carbon sequestration via sinking (yellow-green shades) or GHG emissions mitigation via products (green-blue shades) is cheaper. Sequestration via sinking is generally preferred in locations farthest from port.

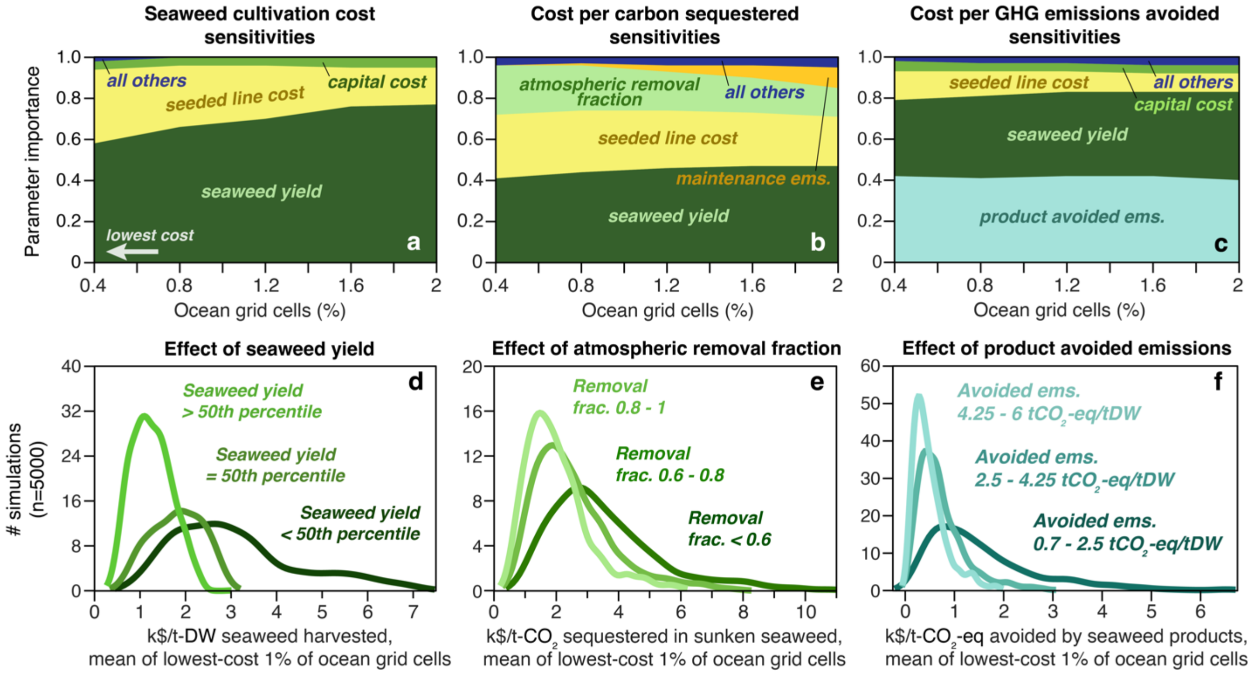

Supplementary Figure 9 | Key cost sensitivities of seaweed production and climate benefits for limited nutrient simulations. Across our Monte Carlo simulations in the 2% of ocean grid cells where costs are lowest, estimated seaweed production cost is especially sensitive to the seaweed yield amount and seeded line cost (**a**), whereas costs of carbon sequestration (**b**) and GHG emissions avoided (**c**) are strongly influenced by the fraction of seaweed carbon that corresponds to an equivalent amount removed from the atmosphere and the assumed emissions avoided by seaweed products, respectively, in addition to seaweed yield and seeded line cost. Panels **d-f** show kernel density plots for the most important parameters in the cheapest 1% ocean areas, showing that the lowest production and climate benefit costs depend upon seaweed yield being at or above the median of potential seaweed yields (**d**), an assumed atmospheric removal fraction of >0.6-0.8 (**e**), and avoided emissions >2.5 tCO_2-eq/tDW (**f**).

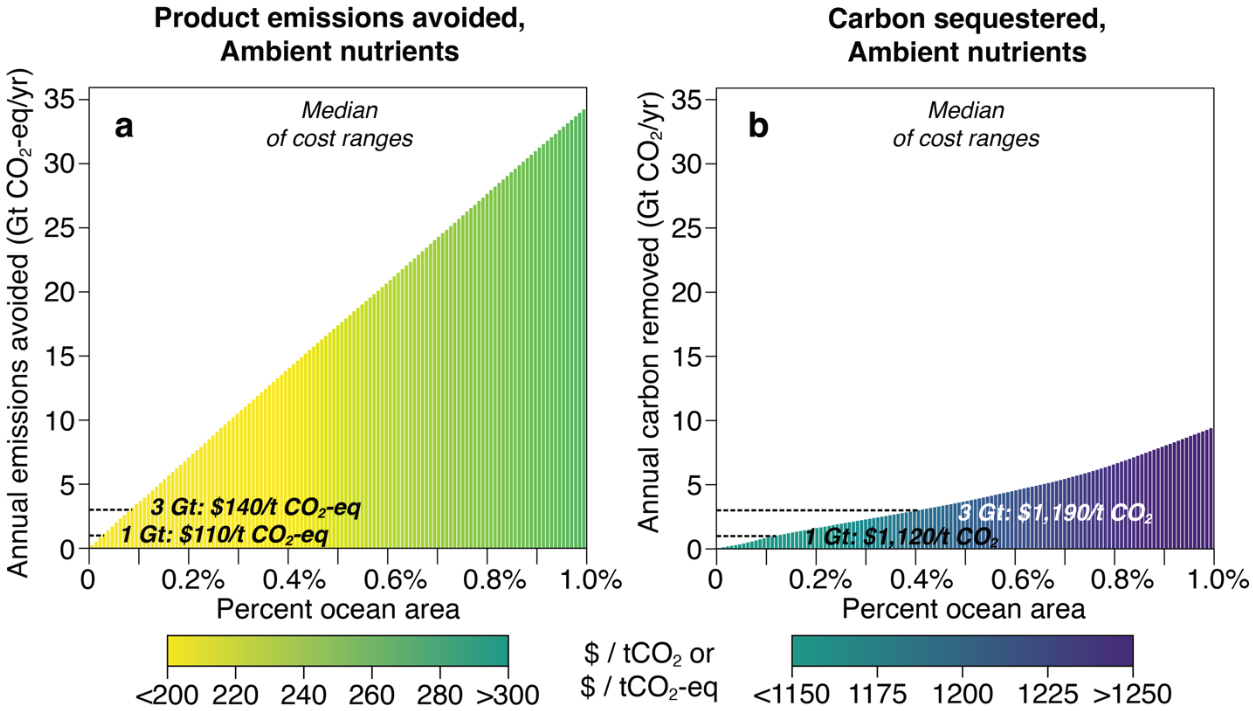

Supplementary Figure 10 | Cumulative potential climate benefits of large-scale seaweed farming using median of cost simulations. Total GHG emissions avoided (**a**) or carbon sequestered (**b**) each year could reach gigaton-scales if seaweed were farmed over large areas of the ocean. Bars show the potential climate benefits as a function of the lowest-cost ocean area (0.1% of ocean area is roughly 360,000 km^2, nearly the area of Germany and 130 times the total area of current seaweed farms), and colors indicate the average cost per tCO_2-eq emissions avoided or tCO_2 sequestered using median net costs from ambient nutrient simulations.

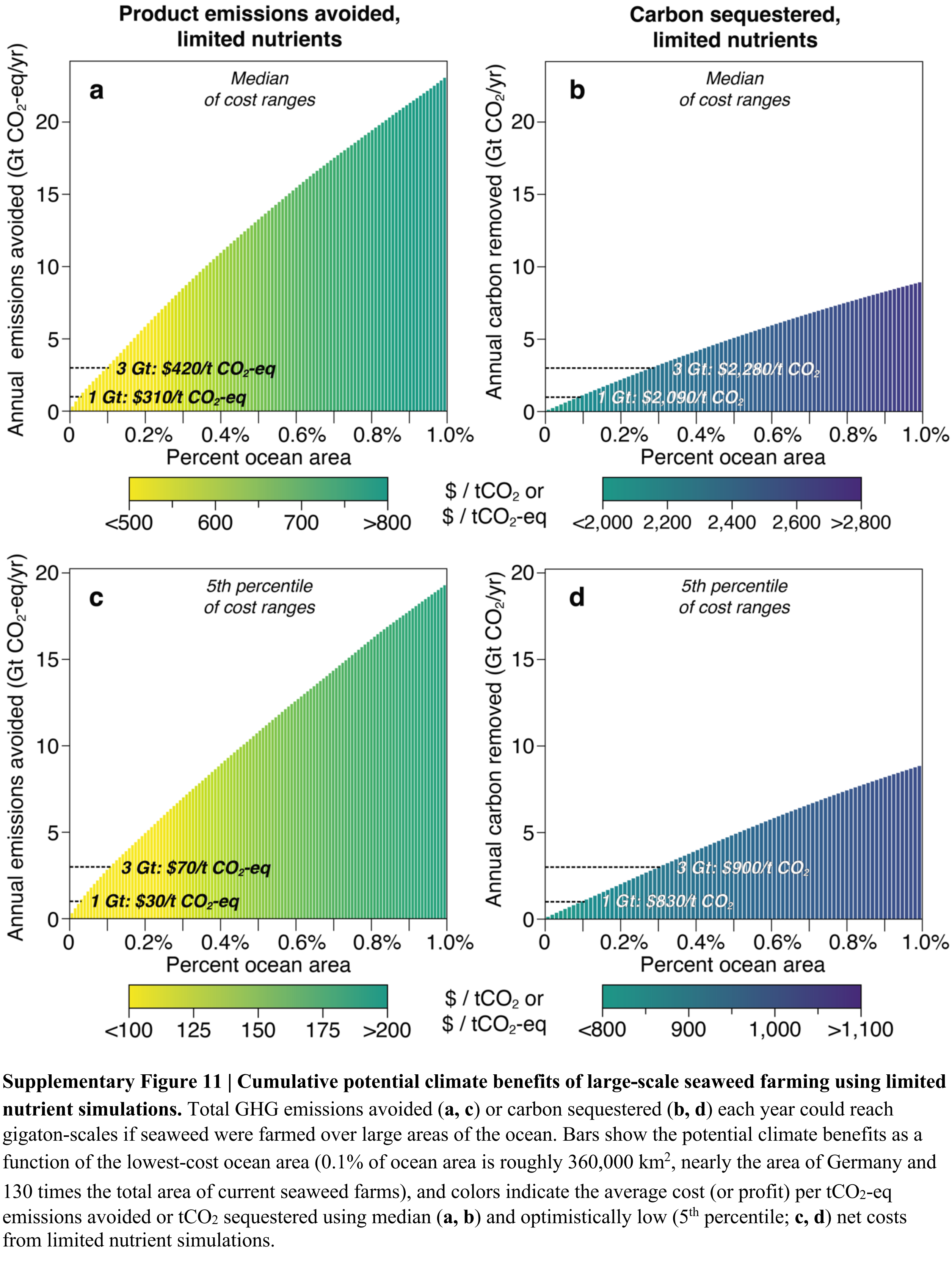

Supplementary Figure 11 | Cumulative potential climate benefits of large-scale seaweed farming using limited nutrient simulations. Total GHG emissions avoided (**a, c**) or carbon sequestered (**b, d**) each year could reach gigaton-scales if seaweed were farmed over large areas of the ocean. Bars show the potential climate benefits as a function of the lowest-cost ocean area (0.1% of ocean area is roughly 360,000 km^2, nearly the area of Germany and 130 times the total area of current seaweed farms), and colors indicate the average cost (or profit) per tCO_2-eq emissions avoided or tCO_2 sequestered using median (**a, b**) and optimistically low (5th percentile; **c, d**) net costs from limited nutrient simulations.

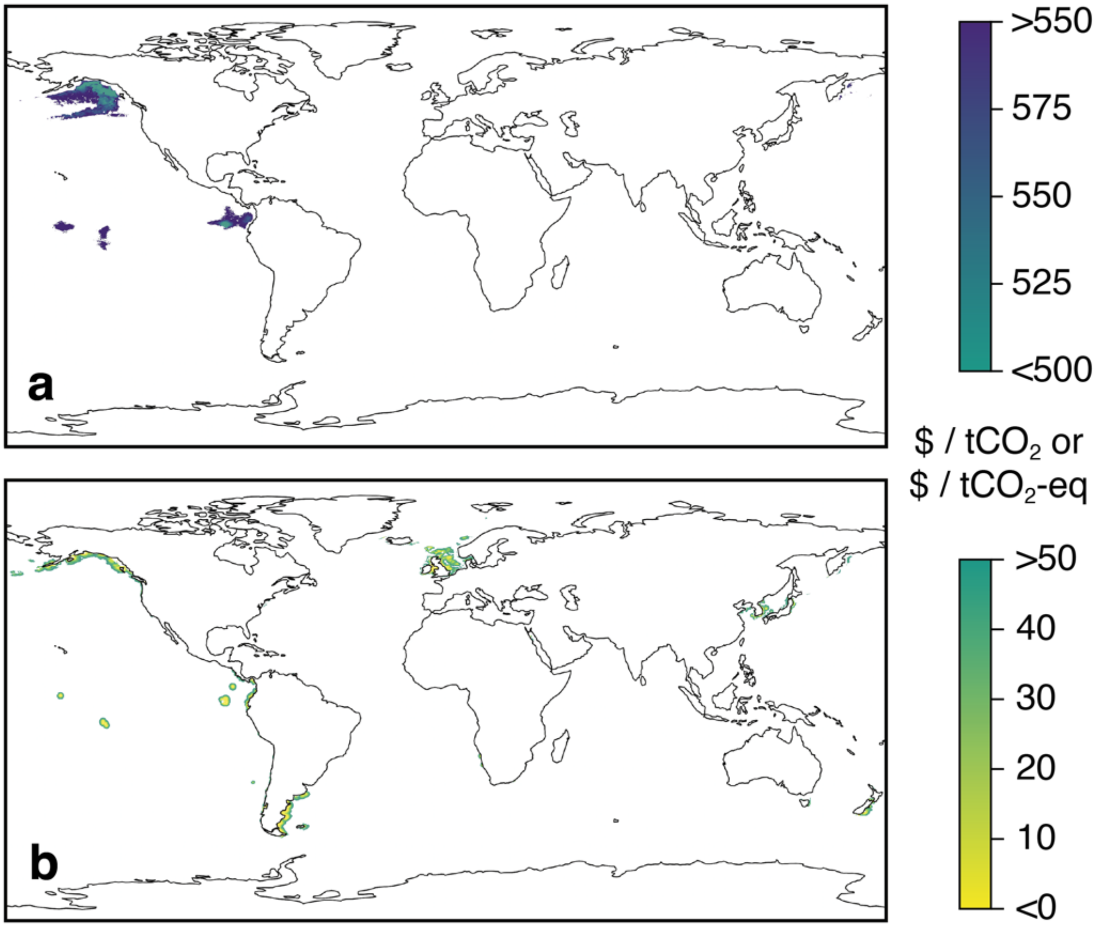

Supplementary Figure 12 | Maps of lowest cost areas in Fig. 4. Maps show lowest-cost areas in the cheapest 1% of seaweed growth area for carbon sequestration (**a**) and GHG emissions avoided (**b**) in the 5th percentile of ambient nutrient simulations, with the color of shaded areas representing the net cost per ton of CO_2 or CO_2-eq.

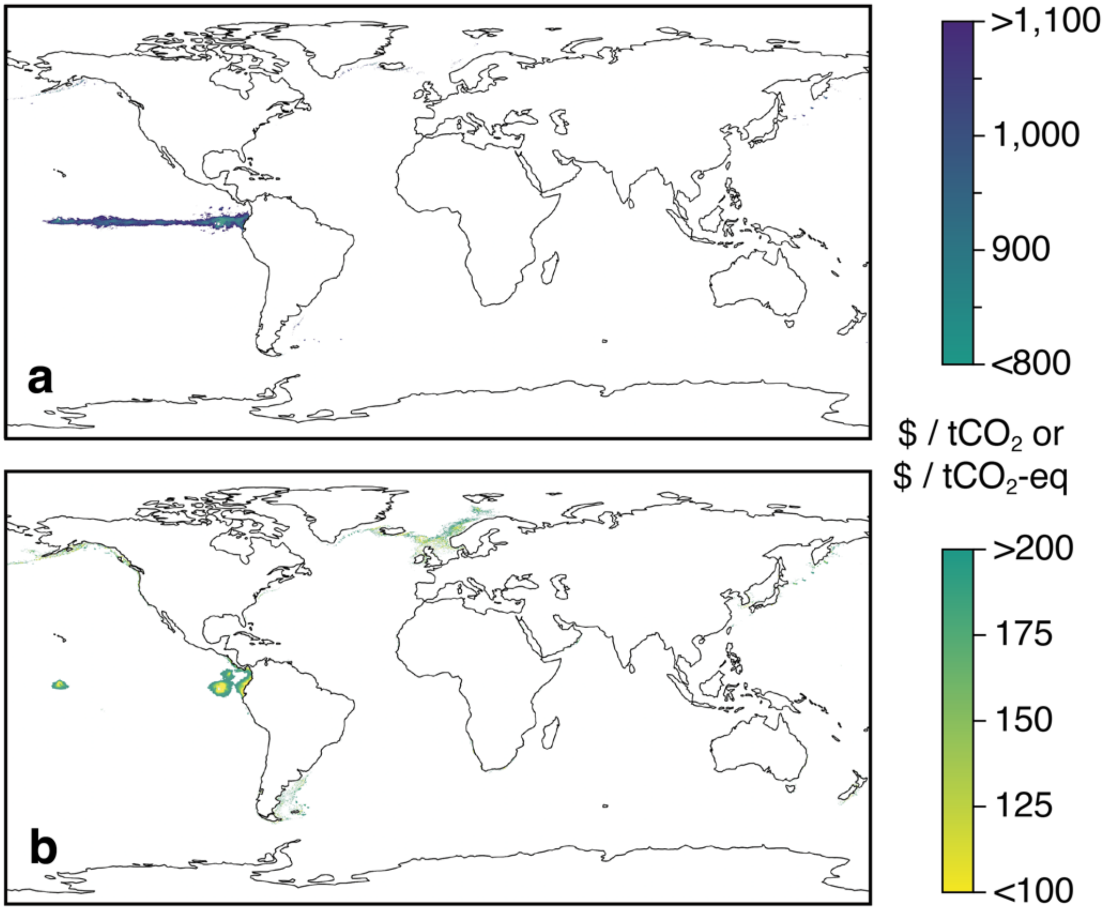

Supplementary Figure 13 | Maps of lowest cost areas in Supplementary Fig. 11c,d. Maps show lowest-cost areas in the cheapest 1% of seaweed growth area for carbon sequestration (**a**) and GHG emissions avoided (**b**) in the 5th percentile of limited nutrient simulations, with the color of shaded areas representing the net cost per ton of CO_2 or CO_2-eq.

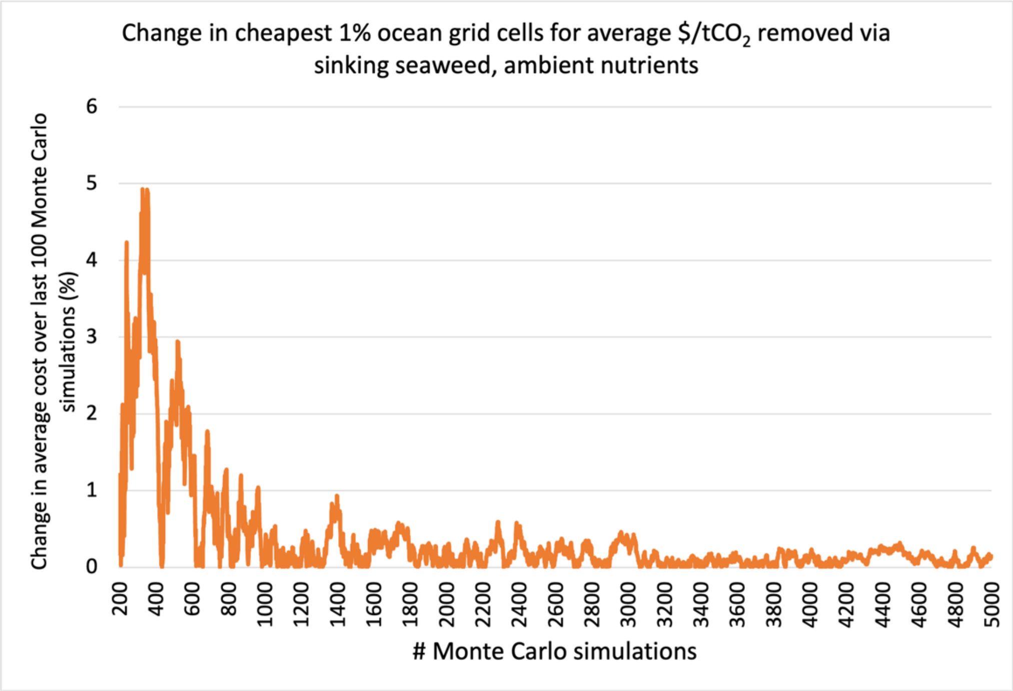

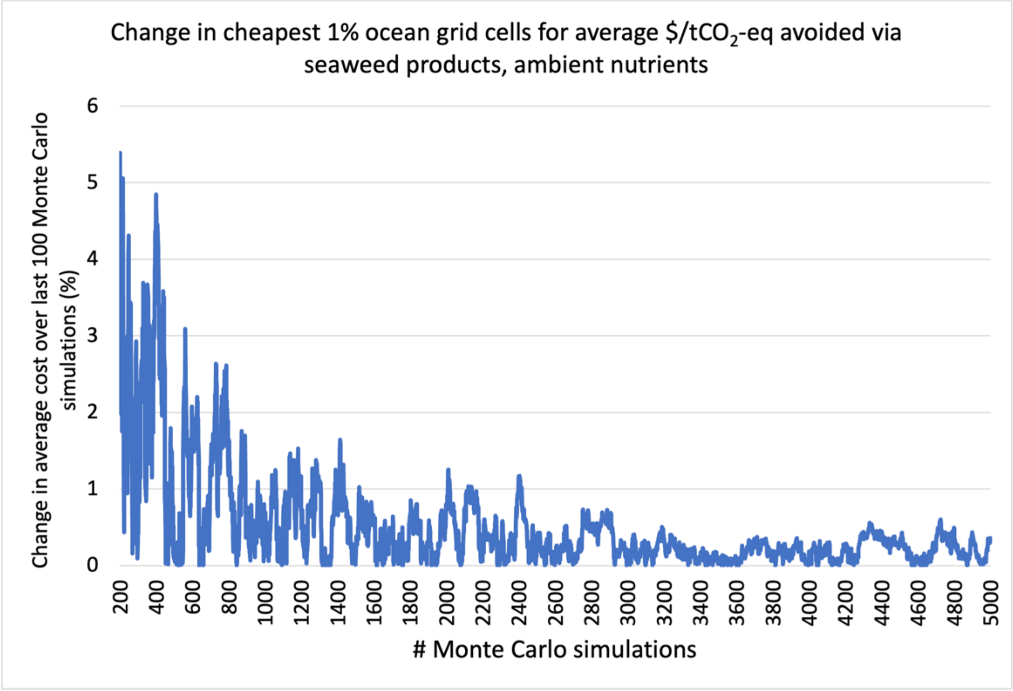

Supplementary Figure 14 | Change in ambient nutrient scenario average costs of CDR (top) and avoided emissions (bottom) with successive Monte Carlo simulations. Monte Carlo simulation number (n) is shown on the x-axis, and the % change in the cheapest 1% ocean grid cells average cost over the previous 100 runs is shown on the y-axis.

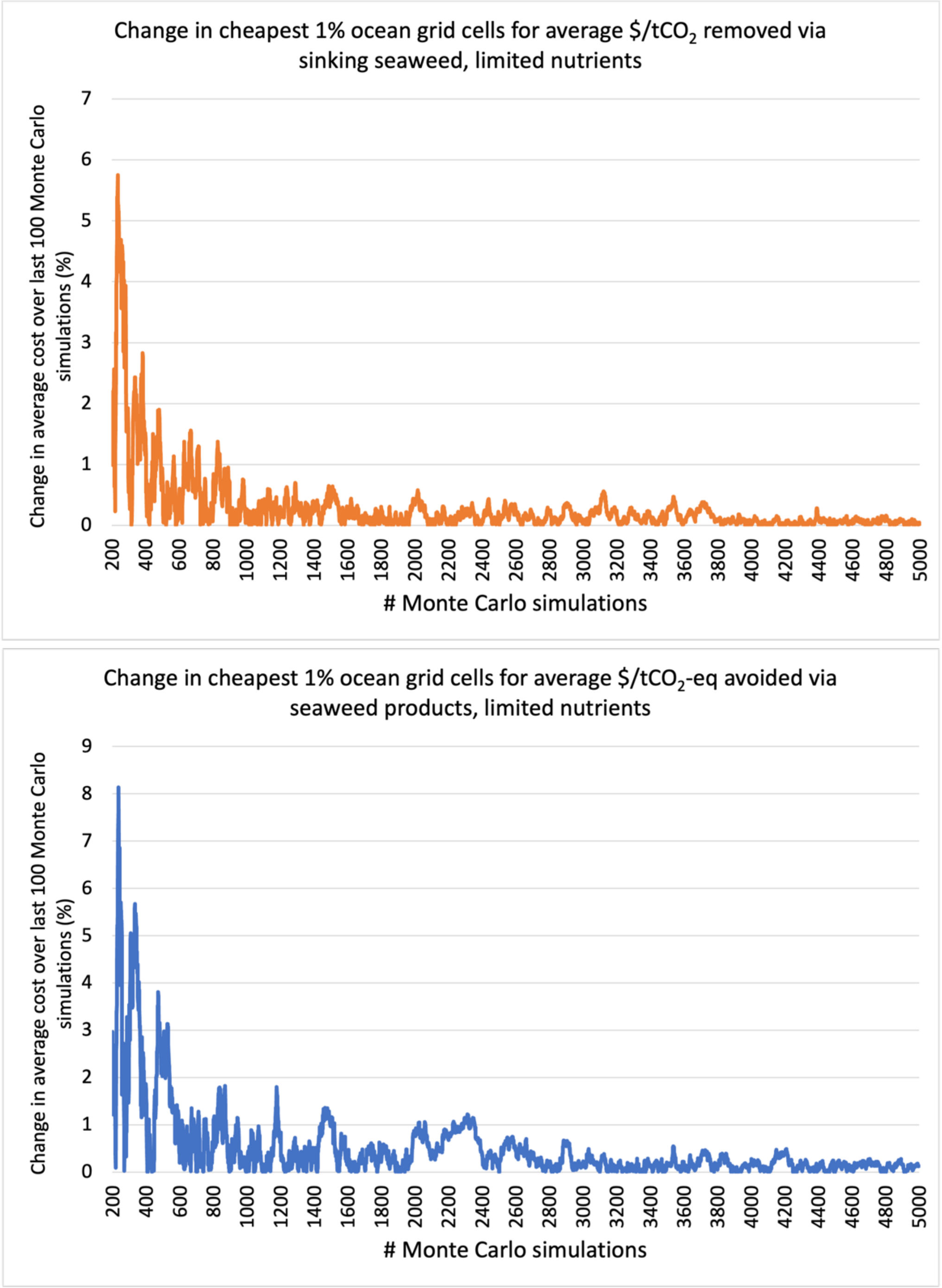

Supplementary Figure 15 | Change in limited nutrient scenario average costs of CDR (top) and avoided emissions (bottom) with successive Monte Carlo simulations. Monte Carlo simulation number (n) is shown on the x-axis, and the % change in the cheapest 1% ocean grid cells average cost over the previous 100 runs is shown on the y-axis.

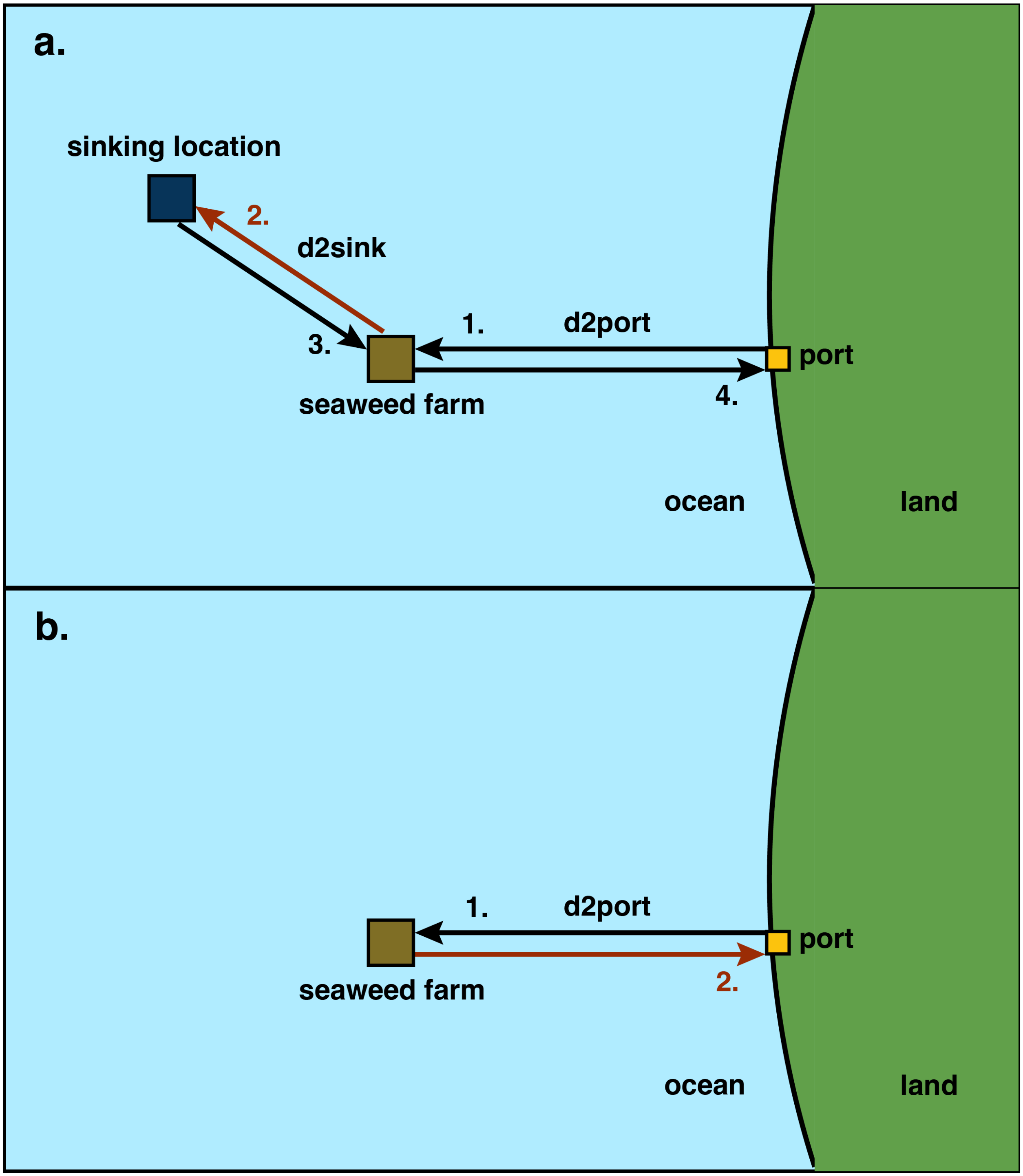

Supplementary Figure 16 | Schematic of model transport framework for carbon sequestration via sinking (a) and avoided GHG emissions via products (b). Arrows indicate direction of transport, numbers next to arrows indicate order of transport steps, and red arrows indicate that harvested seaweed is being transported during that step.

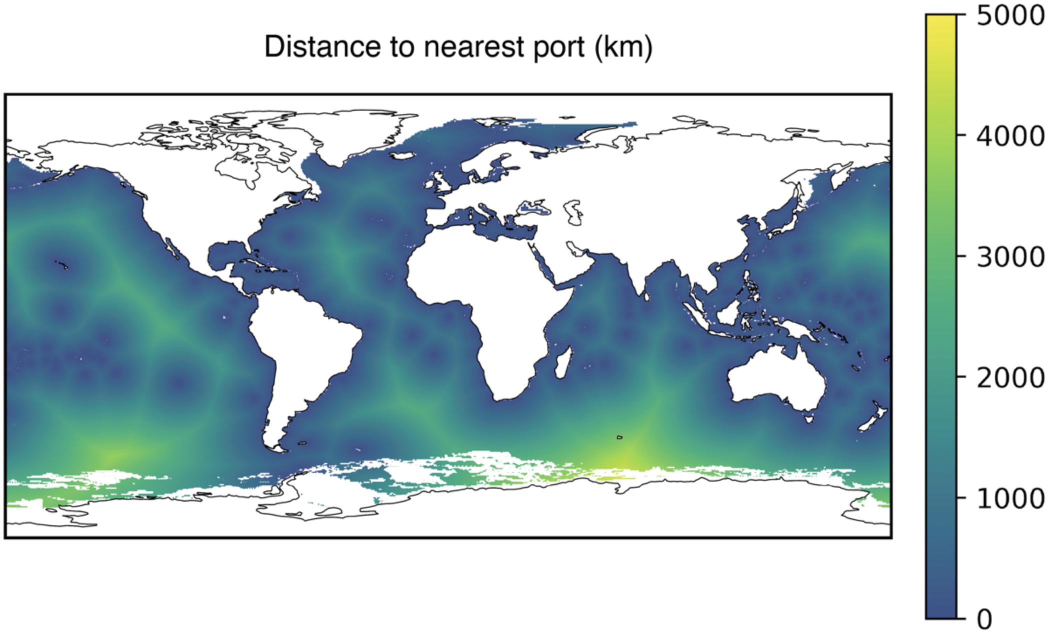

Supplementary Figure 17 | Distance to port. Map shows the distance to the nearest port (km) from every ocean grid cell, interpolated from the Global Fishing Watch Distance from Port V1 dataset [23].

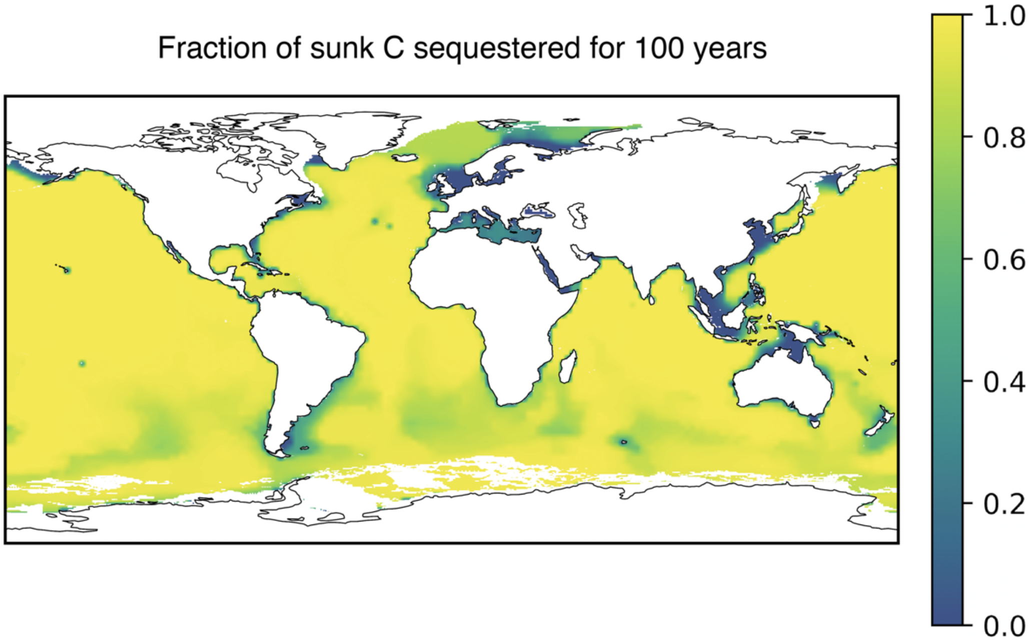

Supplementary Figure 18 | Fraction of deposited carbon sequestered for 100 years. Data from Siegel et al. (2021)[18] interpolated to 1/12-degree grid resolution.

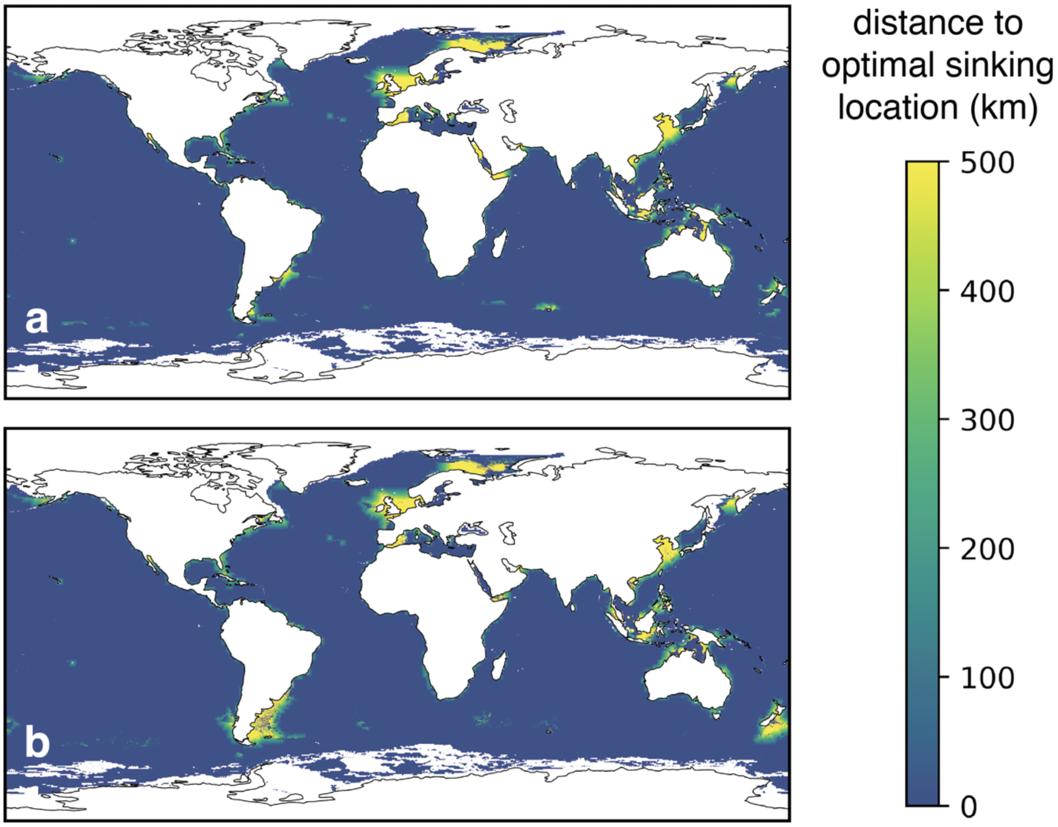

Supplementary Figure 19 | Distance to economically-optimized sinking location. Maps show the shortest ocean distance from each seaweed growth pixel to the location at which the net CO_2 removed is maximized (incl. impacts of both increased sequestration fraction and transport emissions for different potential sinking locations) and the net cost is minimized for median ambient nutrient (**a**) and limited nutrient (**b**) scenarios. See *Methods* for detailed discussion of calculations.

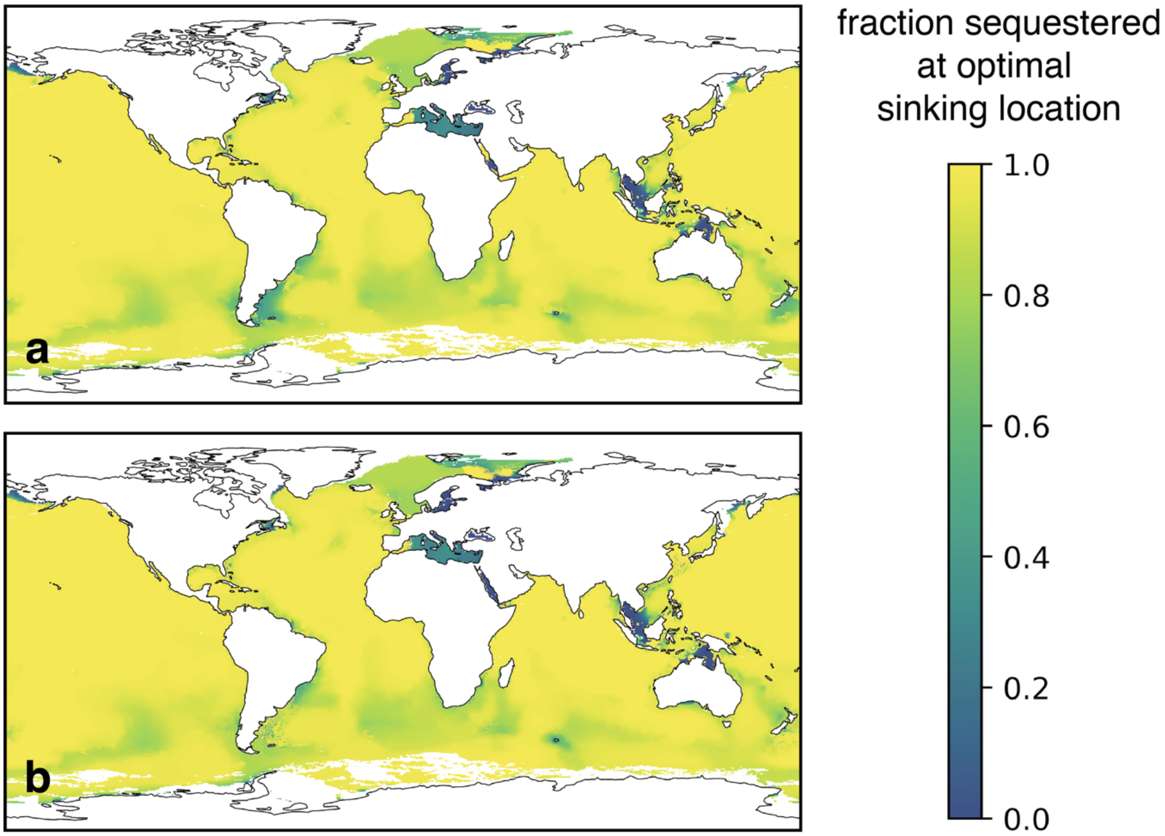

Supplementary Figure 20 | Fraction of deposited carbon sequestered for 100 years with transport to optimal sinking location. After being transported to the optimal sinking location, the fraction sequestered for that location is applied to the grid cell where the seaweed was grown. The resulting adjusted fraction sequestered maps used in our economic calculations are shown for median ambient nutrient (**a**) and limited nutrient (**b**) scenarios.

References

1 van den Burg, S. W. K., van Duijn, A. P., Bartelings, H., van Krimpen, M. M. & Poelman, M. The economic feasibility of seaweed production in the North Sea. *Aquaculture Economics and Management* (2016). https://doi.org:10.1080/13657305.2016.1177859

2 Capron, M. *et al.* *AdjustaDepth TechnoEconomic Analysis for ARPA-E Phase 1 Contract DE-AR0000916* (2020).

3 Correa, T. *et al.* Production and economic assessment of giant kelp *Macrocystis pyrifera* cultivation for abalone feed in the south of Chile. *Aquaculture Research* **47**, 698-707 (2016). https://doi.org:10.1111/are.12529

4 Camus, C., Infante, J. & Buschmann, A. H. Revisiting the economic profitability of giant kelp Macrocystis pyrifera (Ochrophyta) cultivation in Chile. *Aquaculture* (2019). https://doi.org:10.1016/j.aquaculture.2018.12.030

5 Aitken, D., Bulboa, C., Godoy-Faundez, A., Turrion-Gomez, J. L. & Antizar-Ladislao, B. Life cycle assessment of macroalgae cultivation and processing for biofuel production. *Journal of Cleaner Production* **75** (2014). https://doi.org:10.1016/j.jclepro.2014.03.080

6 Johnson, T. Saving Fuel on Your Recreational or Charter Boat. 2-3 (Alaska Sea Grant Marine Advisory Program, 2011).

7 Berger, M., Bopp, L., Ho, D. T. & Kwiatkowski, L. in *EGU General Assembly* (Vienna, Austria, 2022).

8 Harrison, C. S., Long, M. C., Lovenduski, N. S. & Moore, J. K. Mesoscale Effects on Carbon Export: A Global Perspective. *Global Biogeochemical Cycles* **32**, 680-703 (2018). https://doi.org:https://doi.org/10.1002/2017GB005751

9 Bach, L. T. *et al.* Testing the climate intervention potential of ocean afforestation using the Great Atlantic Sargassum Belt. *Nature Communications* **12**, 2556 (2021). https://doi.org:10.1038/s41467-021-22837-2

10 Arzeno-Soltero, I. *et al.* Biophysical potential and uncertainties of global seaweed farming. *preprint retrieved from EarthArXiv* (2022). https://doi.org:https://doi.org/10.31223/X52P8Z

11 *Dried Seaweed Price*, <https://www.alibaba.com/showroom/dried-seaweed-price.html> (2022).

12 USDA. National Weekly Feedstuff Wholesale Prices. (2022).

13 U.S.DOE. *Average Retail Fuel Prices in the United States*, <https://afdc.energy.gov/data/10326> (2022).

14 Buschmann, A. H. *et al.* Seaweed production: overview of the global state of exploitation, farming and emerging research activity. *European Journal of Phycology* **52**, 391-406 (2017). https://doi.org:10.1080/09670262.2017.1365175

15 Roesijadi, G., Jones, S. B., Snowden-Swan, L. J. & Zhu, Y. Macroalgae as a Biomass Feedstock: A Preliminary Analysis. (Pacific Northwest National Laboratory, 2010).

16 Hong, C. *et al.* Global and regional drivers of land-use emissions in 1961–2017. *Nature* (2021). https://doi.org:10.1038/s41586-020-03138-y

17 EIA. (Carbon Dioxide Emissions Coefficients, U.S. Energy Information Administration, 2021).

18 Siegel, D. A., DeVries, T., Doney, S. & Bell, T. Assessing the sequestration time scales of some ocean-based carbon dioxide reduction strategies. *Environmental Research Letters* (2021). https://doi.org:10.1088/1748-9326/ac0be0

19 Yu, J., Zhang, S., Yang, W., Xin, Y. & Gao, H. Design and application of buoy single point mooring system with electro-optical-mechanical (EOM) cable. *Journal of Marine Science and Engineering* **8** (2020). https://doi.org:10.3390/JMSE8090672

20 Yim, S. C. S., Nakhata, T., Bartel, W. A. & Huang, E. T. Coupled nonlinear barge motions, Part I: Deterministic models development, identification and calibration. *Journal of Offshore Mechanics and Arctic Engineering* **127** (2005). https://doi.org:10.1115/1.1854700

21 Duarte, C. M. Nutrient concentration of aquatic plants: Patterns across species. *Limnology and Oceanography* (1992). https://doi.org:10.4319/lo.1992.37.4.0882

22 USDA. *Seaweed, dried*, <https://fdc.nal.usda.gov/fdc-app.html#/food-details/1103574/nutrients> (2020).

23 Global Fishing Watch. (Distance from Port V1, 2020).
