# Supplementary material for: Economic and biophysical limits to seaweed farming for climate change mitigation
Source: Nat Plants. 2022 Dec 23;9(1):45–57. doi: 10.1038/s41477-022-01305-9 (PMC9873559; doi:10.1038/s41477-022-01305-9)
Supplement: Supplementary file 2 — Reporting Summary [file 41477_2022_1305_MOESM2_ESM.pdf]

nature portfolio

Corresponding author(s): Julianne DeAngelo, Steven J. Davis

Last updated by author(s): Oct 28, 2022

Reporting Summary

Nature Portfolio wishes to improve the reproducibility of the work that we publish. This form provides structure for consistency and transparency in reporting. For further information on Nature Portfolio policies, see our Editorial Policies and the Editorial Policy Checklist.

Statistics

For all statistical analyses, confirm that the following items are present in the figure legend, table legend, main text, or Methods section.

n/a	Confirmed	
[ ]	[x]	The exact sample size (*n*) for each experimental group/condition, given as a discrete number and unit of measurement
[ ]	[x]	A statement on whether measurements were taken from distinct samples or whether the same sample was measured repeatedly
[x]	[ ]	The statistical test(s) used AND whether they are one- or two-sided<br>*Only common tests should be described solely by name; describe more complex techniques in the Methods section.*
[ ]	[x]	A description of all covariates tested
[ ]	[x]	A description of any assumptions or corrections, such as tests of normality and adjustment for multiple comparisons
[ ]	[x]	A full description of the statistical parameters including central tendency (e.g. means) or other basic estimates (e.g. regression coefficient) AND variation (e.g. standard deviation) or associated estimates of uncertainty (e.g. confidence intervals)
[x]	[ ]	For null hypothesis testing, the test statistic (e.g. *F*, *t*, *r*) with confidence intervals, effect sizes, degrees of freedom and *P* value noted<br>*Give P values as exact values whenever suitable.*
[ ]	[x]	For Bayesian analysis, information on the choice of priors and Markov chain Monte Carlo settings
[ ]	[x]	For hierarchical and complex designs, identification of the appropriate level for tests and full reporting of outcomes
[x]	[ ]	Estimates of effect sizes (e.g. Cohen's *d*, Pearson's *r*), indicating how they were calculated

Our web collection on statistics for biologists contains articles on many of the points above.

Software and code

Policy information about availability of computer code

Data collection	Model code was developed by B. Saenz, S. J. Davis, and J. DeAngelo. Python version 3.9. Code available via Zenodo: https://doi.org/10.5281/zenodo.7262015.
Data analysis	Data analysis code was developed by B. Saenz and J. DeAngelo using Python 3.9. Full code available via Zenodo: https://doi.org/10.5281/zenodo.7262015. Details: Code for Random Forest analysis was modified from open-source "Random Forest in Python" tutorial by Towards Data Science. Distance to the optimal sinking point was calculated using a weighted distance transform (path-finding algorithm, modified from code by Omar Richardson (2020). Citation: Richardson, O. weighted_distance_transform, <https://github.com/0mar/weighted-distance-transform> (2020).

For manuscripts utilizing custom algorithms or software that are central to the research but not yet described in published literature, software must be made available to editors and reviewers. We strongly encourage code deposition in a community repository (e.g. GitHub). See the Nature Portfolio guidelines for submitting code & software for further information.

Data

Policy information about availability of data

All manuscripts must include a data availability statement. This statement should provide the following information, where applicable:

- Accession codes, unique identifiers, or web links for publicly available datasets
- A description of any restrictions on data availability
- For clinical datasets or third party data, please ensure that the statement adheres to our policy

Data is available to download from Dryad: https://doi.org/10.7280/D13H59 .

Field-specific reporting

Please select the one below that is the best fit for your research. If you are not sure, read the appropriate sections before making your selection.

☐ Life sciences ☐ Behavioural & social sciences ☒ Ecological, evolutionary & environmental sciences

For a reference copy of the document with all sections, see nature.com/documents/nr-reporting-summary-flat.pdf

Ecological, evolutionary & environmental sciences study design

All studies must disclose on these points even when the disclosure is negative.

Study description	We developed a technoeconomic model of costs and net emissions associated with farming seaweed for climate benefits at scales relevant to the global carbon budget. Given the large uncertainty in the technoeconomic model parameters, we performed a Monte Carlo analysis, sampling uniformly across parameter ranges to produce 5,000 unique simulations of cost per ton of CO2 sequestered or avoided in any given location for each seaweed nutrient scenario. We then assessed the 5th, 25th, 50th, 75th, and 95th percentile Monte Carlo results globally, as well as the lowest-cost 1% areas for each scenario. Finally, we performed a LightGBM analysis of model parameters for the lowest-cost regions to determine variable importance across the Monte Carlo simulations.
Research sample	* Seaweed biomass harvested: We use spatially-explicit data for seaweed harvested globally under both ambient and flux-limited nutrient scenarios from the G-MACMODS seaweed growth model, presented in Arzeno-Soltero et al. (https://doi.org:https://doi.org/10.31223/X52P8Z).<br>* Fraction of deposited carbon sequestered for 100 years at seafloor depth: We use data from Siegel et al. (2021) interpolated to our 1/12-degree grid resolution.<br>* Distance to nearest port: We use the Distance from Port V1 dataset from Global Fishing Watch (https://globalfishingwatch.org/data-download/datasets/public-distance-from-port-v1) interpolated to our 1/12-degree grid resolution.<br>* Significant wave height: We use data for annually-averaged significant wave height from the European Center for Medium-range Weather Forecasts (ECMWF) interpolated to our 1/12-degree grid resolution.<br>* Ocean depth: We use data from the General Bathymetric Chart of the Oceans (GEBCO).<br>* Shipping lanes: We use data of Automatic Identification System (AIS) signal count per ocean grid cell, interpolated to our 1/12-degree grid resolution. We define a major shipping lane grid cell as any cell with >2.25 x 10^8 AIS signals, a threshold that encompasses most major trans-Pacific and trans-Atlantic shipping lanes as well as major shipping lanes in the Indian Ocean, North Sea, and coastal routes worldwide.<br>* Marine Protected Areas (MPAs): We use data from the World Database on Protected Areas (WDPA) and define a MPA as any ocean WDPA >20 km^2.
Sampling strategy	Due to the lack of existing data for the technoecomoic variables in our model, we assumed a uniform distribtion across uncertainty ranges and sampled randomly across those ranges for each variable. Seaweed yield was sampled from a normal distribution according to uncertainty analysis from Arzeno-Soltero et al. (https://doi.org:https://doi.org/10.31223/X52P8Z).
Data collection	Data files were recorded and saved as netCDF files throughout model runs, and statistical metadata was saved as .csv files. Data was compiled and analyzed by J. DeAngelo, using code developed by B. Saenz and J. DeAngelo (Python version 3.9).
Timing and spatial scale	We produced 5,000 simulations for each seaweed nutrient scenario for our Monte Carlo analysis. Each simulation represents the potential cost per ton of carbon sequestered or avoided by either growing and sinking seaweed or using seaweed to replace emissions-intensive products. These costs assume that the maximum seaweed biomass could be grown annually in the G-MACMODS seaweed growth model (Arzeno-Soltero et al., https://doi.org:https://doi.org/10.31223/X52P8Z). G-MACMODS is a global, spatially-explicit seaweed growth model; our technoeconomic model is also global and spatially-explicit in assessing the costs and net emissions associated with farming the seaweed represented by G-MACMODS.
Data exclusions	We excluded negative cost values from our LightGBM analysis of the lowest-cost areas from Monte Carlo simulations, because there were too few negative values for the algorithm to be statistically robust at predicting values below zero.
Reproducibility	Attempts to repeat the Monte Carlo and LightGBM analyses were sucessful.
Randomization	Training and test groups for LightGBM analyses were sampled randomly using 100 decision trees, with random state set to 42.
Blinding	Blinding was not relevant to our study.

Did the study involve field work? ☐ Yes ☒ No

Reporting for specific materials, systems and methods

We require information from authors about some types of materials, experimental systems and methods used in many studies. Here, indicate whether each material, system or method listed is relevant to your study. If you are not sure if a list item applies to your research, read the appropriate section before selecting a response.

Materials & experimental systems

n/a	Involved in the study
☒	☐ Antibodies
☒	☐ Eukaryotic cell lines
☒	☐ Palaeontology and archaeology
☒	☐ Animals and other organisms
☒	☐ Human research participants
☒	☐ Clinical data
☒	☐ Dual use research of concern

Methods

n/a	Involved in the study
☒	☐ ChIP-seq
☒	☐ Flow cytometry
☒	☐ MRI-based neuroimaging
